# A scoping review of emotional contagion research with human subjects: identifying common trends of previous research and potential areas for future research

**DOI:** 10.3389/fpsyg.2025.1573375

**Published:** 2025-05-30

**Authors:** Barret Michalec, Chad E. Forbes, Kevin Pardon, Bella Ayala, Diego Guevara Beltran, Clarice Douille, Kaitlyn Felix, Samantha Gnall, Michael Hoenack, Brooke McKeever, Daniel Nguyen, Nicole Piemonte, Sarah Portle

**Affiliations:** ^1^Edson College, Arizona State University, Phoenix, AZ, United States; ^2^Arizona State University Library, Arizona State University, Tempe, AZ, United States; ^3^Creighton University School of Medicine, Phoenix, AZ, United States; ^4^Department of Psychology, University of Arizona, Tucson, AZ, United States; ^5^College of Health Solutions, Arizona State University, Tempe, AZ, United States; ^6^Department of Psychology, Florida Atlantic University, Boca Raton, FL, United States; ^7^College of Liberal Arts and Sciences, Arizona State University, Tempe, AZ, United States

**Keywords:** emotional contagion, human subjects, scoping review, emotional mimicry, methodology, conceptualization

## Abstract

**Introduction:**

Emotional contagion (EC) involves the automatic mimicry and synchronization of expressions, vocalizations, and movements, resulting in emotional alignment between individuals. Despite consistent scholastic explorations of the various nuances and tenets associated with emotional contagion processes and outcomes, there has yet to be a thorough review of human subjects-based emotional contagion research.

**Methods:**

This review examines human subjects EC research trends, analyzing 277 articles (published from 1992 to 2022) to identify common conceptualizations, triggers, and measurement methods.

**Results:**

Analyses indicated that Hatfield et al.’s classic conceptualization is the most cited, and common triggers include facial expressions in images and videos, and real-time interactions - though many studies did not stimulate EC. While many studies did utilize validated EC scales, about 28% of the studies reviewed used non-validated questions to measure EC. Moreover, the EC research reviewed heavily relies on college-aged, predominantly white participants, indicating a need for more diverse samples.

**Discussion:**

Future EC research should explore processes and nuances associated with EC among older adults, minoritized groups, and diverse contexts (e.g., healthcare, schools), using novel triggers and multiple measurement methods.

## Introduction

Emotional contagion (EC) is defined theoretically as “…the tendency to automatically mimic and synchronize facial expressions, vocalizations, postures, and movements with those of another person and, consequently, to converge emotionally” (Hatfield et al., 1992). Since its introduction to the literature, EC has had a broad influence on a number of fields within and outside of psychology given its relevance or applicability to a number of core psychological processes (Hatfield et al., 2014; Herrando and Constantinides, 2021; Pérez-Manrique and Gomila, 2022). EC plays a significant role in shaping social interactions, group dynamics, and even decision-making processes (Parkinson and Simons, 2009; Bosse et al., 2013). For example, Hatfield et al. (2009) detail how EC contributes to the synchronization of emotional states during interpersonal encounters, facilitating rapport and emotional alignment. Their work supports the idea that EC undergirds basic processes of social bonding and affiliative behavior, making it central to understanding how people form connections and interpret each other’s emotions in real-time. In the area of group dynamics, Barsade et al. (2018) have shown that EC significantly shapes emotional climates within organizations. Their research demonstrates how the affective tone of a single team member can “spread” and influence group cohesion, trust, and overall performance. This highlights the relevance of EC for leadership, teamwork, and workplace culture—areas critical to organizational psychology and management studies. Additionally, Parkinson and Simons (2009) explored how EC influences individual judgments in emotionally charged group settings, finding that emotional states shared via contagion can bias cognitive processing and risk perception. Similarly, Bosse et al. (2013) used agent-based modeling to show how EC affects decision trajectories in simulated environments, emphasizing its role in both intuitive and deliberative reasoning processes.

Moreover, EC has also been argued to function as an essential element of the empathy experience (Hatfield et al., 2009; de Waal and Preston, 2017). Whereas empathy represents the totality of the cognitive and affective experience of another’s emotional state (either affectively or cognitively), EC represents the initial “catching” (to some degree) of the other’s emotion. Moreover, EC is distinct from sympathy in that sympathy reflects “feeling *for*” and EC, as part of the empathy experience, reflects “feeling *with*” (Michalec and Hafferty, 2022). Although extensive research explores facets and outcomes related to the empathy experience, less is known regarding the processes and mechanisms of EC, particularly in humans. Given that scholars have highlighted the primitive, evolutionary nature of EC (de Waal and Preston, 2017), studying trends in research on EC in human subjects offers invaluable insights into the intricate dynamics of interpersonal communication and emotional influence. It also provides a deeper understanding of how emotions spread across individuals and impact various aspects of their lives.

It is clear that EC is a foundational mechanism in the human emotional experience—shaping various social and psychological processes from how decisions are made, to how individuals connect and groups function. And yet, whereas there have been recent reviews of EC research (Herrando and Constantinides, 2021; Hatfield et al., 2014; Barsade et al., 2018; Banerjee and Srivastava, 2019, Goldenberg and Gross, 2020; among others), a majority of these reviews have been general in nature (i.e., not scoping or systematic) and/or have been focused on showcasing the EC research particular to a specific sub-field (e.g., marketing, organizational behavior, digital media, etc.). Moreover, Pérez-Manrique and Gomila (2022) provide a robust and comprehensive review of EC research—focused on *nonhuman* animals. Thus, this particular scoping review offers a differing targeted focus, the “what,” “how,” and “who” of EC research with human subjects by addressing the following questions: (a) what are the measures, triggers, and conceptualization of EC?, (b) what common trends exist regarding EC research?, and (c) what commonalities exist among/within EC research on human subjects? More specifically, we identify common trends and potential areas of future EC research regarding the conceptualization, triggers, and measures of EC. We also provide insight on the general topics and the samples utilized within recent EC research with human subjects. Our goal with this extensive scoping review is to not only outline the commonalities within EC research on human subjects conducted over the last three decades (i.e., where the research *has been*) but also point researchers to fertile aspects of EC research that has received minimal attention thus far, in order to spark scholarly curiosity and push EC research into new arenas.

By examining the frameworks employed in earlier studies, we can identify the key dimensions that have shaped our comprehension of EC over time. This historical perspective enables the field to build upon established knowledge, refine existing theories, and explore novel avenues of inquiry. Additionally, an analysis of past conceptualizations highlights gaps, inconsistencies, and potential biases in the literature, fostering a more robust and accurate portrayal of how emotions are transmitted, received, and transformed among individuals.

Exploring the “triggers” and measures of EC with human subjects (i.e., the “how”) holds significant importance, as it offers insights into the catalysts that initiate and propagate emotional influence within social interactions. Investigating the diverse triggers whether facial expressions, verbal cues, situational contexts, or other mechanisms sheds light on how emotions are transmitted and adopted. It also helps identify the parameters and limitations of extant EC research. Examining exactly how EC has been both operationalized and quantified or measured to date can help elucidate what exactly EC “is” and whether the conclusions derived from these studies (sometimes more sensationalized) are warranted and/or warrant caution with respect to internal and external validity of findings. Researchers can accurately quantify its effects and understand its effect on cognitive, physiological, and behavioral levels by identifying reliable measures and methodologies to assess EC (Goldenberg and Gross, 2020; Herrando and Constantinides, 2021). This knowledge not only enhances our comprehension of the underlying processes but also paves the way for developing targeted interventions that leverage EC to promote positive social dynamics, well-being, and effective communication strategies.

Examining samples used in EC research with human subjects (i.e., the “who”) is of utmost importance as it ensures the generalizability and applicability of findings to diverse populations and contexts. By scrutinizing the characteristics of the samples, including factors such as age, cultural background, gender, and psychological traits, researchers can discern the extent to which the observed EC effects are consistent across different groups (Barsade et al., 2018; Doherty, 1997). This analysis not only aids in understanding potential variations in susceptibility to emotional influence but also enables the identification of factors that may moderate or mediate the transmission of emotions. Ultimately, a comprehensive examination of sample demographics enhances the validity and robustness of EC research, enabling researchers to offer relevant and valuable insights to a broad spectrum of individuals and settings.

Scoping reviews of research are crucial, as they assess the breadth, depth, and nature of existing literature on a topic and can be used to map key concepts as well as identify gaps and avenues for further research (Arksey and O’Malley, 2005). Scoping reviews differ from systematic reviews in that systematic reviews aim to answer a specific research question (often about effectiveness or outcomes)—they are typically grounded in critical appraisal to evaluate the quality of evidence. Because this review is aimed more broadly at identifying common conceptualizations, triggers, and measurement methods, a scoping review is more appropriate. Similarly, unlike narrative literature reviews that might lack a structured approach, scoping reviews employ a rigorous methodological framework that involves identifying and mapping key concepts, research gaps, and various study designs (Arksey and O’Malley, 2005). Moreover, scoping reviews offer a transparent framework for evaluating various sources, ensuring a more robust overview of the field/topic while minimizing bias (Arksey and O’Malley, 2005). Delving into the trends of EC research with human subjects through a scoping review allows us to unravel the mechanisms through which emotions are transmitted between individuals and where the research has and *has not* been. More specifically, understanding how scholars have conceptualized EC, the samples they have utilized, and how researchers trigger and then measure EC among human subjects provides a necessary foundation for future work in this arena. This foundation, in turn, spotlights commonly utilized strategies and fruitful new directions to expand research on EC (i.e., new populations, new measurement mechanisms, novel emotions, etc.). Overall, examining the trends of EC research holds immense value in deciphering the intricate web of human emotions, fostering healthier relationships, improving mental health outcomes, and shaping the future of human interaction in a technologically driven era.

## Methods

### Search strategy

**Figure 1:**
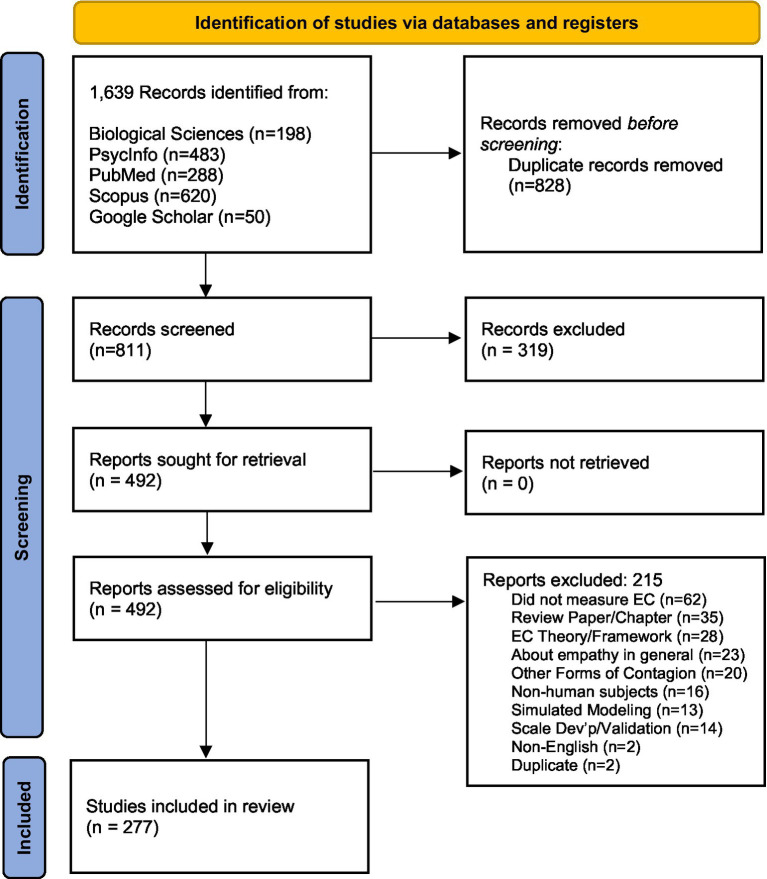
PRISMA diagram of search strategy.

The search strategy for this scoping review was developed by the team’s health sciences librarian with searches conducted in the following databases: *PubMed*, *Scopus*, *PsycINFO,* and *Biological Sciences*. These four databases were selected due to their broad scope and inclusion of many articles published in relevant fields such as behavioral sciences, health sciences, and life sciences. *Google Scholar* was also searched but limited to the first 50 results as a secondary strategy to ensure no vital articles or any particularly relevant pieces of grey literature were missed. Final searches were run between February 24th and March 3rd, 2022, and the results were limited to the English language. The search results were also limited to articles published after January 01, 1992. This particular date was selected as the “start” as it marks the seminal EC work by Hatfield et al. (1992). The search included multiple keywords that combined the concepts of “emotional contagion” AND “measurement” to provide an extensive yet manageable number of results. The idea of “animals” was excluded from the results through the use of the NOT Boolean operator due to this review only focusing on EC related to humans and to decrease the number of irrelevant articles required to be screened by the research team. A detailed description of the search for each database is presented in Appendix A, and Figure 1 features the PRISMA flow diagram displaying the number of records identified, screened, and included in the review.

### Eligibility criteria

Papers were included in this scoping review if they discussed, measured, or were related in any way to the concept of EC in humans. Articles that explored “emotional mimicry” and/or “facial mimicry” were also included in the review as the prominent concept, as offered by Hatfield et al. (1992) does include the notion of “mimicry.”

Papers were excluded from this scoping review if they were:

1. Studies on/with animals, computer-based simulations, model building, or theoretical extrapolations/discussions.
2. Review articles, editorials, commentaries, response papers, books, book chapters, or duplicate articles.
3. Studies that did not explore or measure EC *specifically,* such as examining emotional reactions to stimuli, or empathy in the general sense.
4. Studies on yawn, pain, or burnout contagion.
5. Studies that were not in English.

### Screening and data extraction

The first step in the screening and data extraction process was to examine the abstracts of the initial 811 papers and exclude any articles that did not meet our eligibility criteria. Two research team members (BM & DGB) completed this first screening level and excluded an additional 319 articles (based on the exclusion criteria listed above), leaving 492 articles for full-text screening. If there was disagreement between the two team members during this stage, the whole article was retrieved for full review to confirm inclusion or exclusion eligibility.

The next step in the process was establishing inter-coder reliability (ICR) among the team members. In Phase 1 of establishing ICR, each team member received the same five articles from the review library. Team members were instructed to read each article and identify the *Primary Area/Topic of Research* of the article, the *Conceptualization* utilized for EC (if any), the *“Triggers”/Stimuli* used to initiate the EC process (if any), how EC was *Measured*, and *Characteristics of the Sample* (specifically race, gender, and age, if reported) featured within the study/studies. These categories of analysis were identified collaboratively by the research team as essential to making distinctions among and between the articles and for reporting the key findings of the EC literature review to the broader field. A table was provided to each team member to record their analysis of the articles. Whereas, *Primary Area/Topic of Research* and *Characteristics of the Sample* were limited to one specific entry, the team was instructed to record as many *Conceptualizations*, *“Triggers”/Stimuli*, and *Measures* as were featured in the article. Team members were also required to identify if any article was to be excluded as it did not fall within the inclusion criteria. After reviewing the five articles, team members’ tables were examined to ensure that each team member was identifying the same aspects of the study for each category. Any discrepancies between the team members’ tables were discussed until a consensus was reached. Within Phase 1 of establishing ICR, the team members were in agreement for 95% of the aspects of the articles for each of the categories (only four discrepancies had to be remedied).

Similarly, in Phase 2 of establishing ICR, each team member was provided a new set of the same five articles and told to follow the exact instructions from Phase 1. Phase 2 resulted in 100% team member agreement, with 0 discrepancies. In turn, as it was deemed that ICR had been “met,” following Phase 2, team members were provided a sub-set of the remaining 492 articles and told to follow the exact analysis instructions with those particular articles.

A total of 215 reports were removed from the data set during the full-text review stage of the screening and analysis process. Following the same exclusion criteria as presented above, reports were removed because the study did not measure EC specifically—rather, it examined emotional reactions, emotional regulation, and emotional responses or simply did not mention EC until the Discussion section of the report (62). Similarly, reports that utilized EC theory to frame their approach but did not measure EC in their study were removed (28). Twenty-three reports were removed during this stage because the studies were more about empathy in general (rather than EC specifically)—often, these studies were testing empathy-oriented scales and noting the role of EC within the empathy experience. Other categories of the papers removed during this stage include: review papers, book chapters (35), studies on other forms of contagion (e.g., burnout, yawn, pain, etc.) (20), studies with non-human subjects (14), reports featuring simulated model building/testing (13), non-English articles (2) and duplicates (2). Additionally, during this full-article review stage, another criterion for exclusion was deemed necessary—studies that focused on (sub)scale development or (sub)scale validation were excluded (16). As outlined in Figure 1, the total number of articles remaining after screening and analysis was 277. Appendix B lists the references of the 277 articles included in the scoping review. A table showcasing the coding categories for each of the 277 articles included in the scoping review is available upon request.

## Results

### Trends of EC research with human subjects

Table 1 displays the three most common trends and potential areas for future research found in the literature about the research questions, research interests, or focus areas of the review: *Areas/Topics of Research, Conceptualization(s)*, *Triggers/Stimuli*, *Measures*, and *Sample Characteristics* (i.e., who is being studied). The “Most Common Trends” are the aspects of research most frequently identified by the team per each specific category. Table 1 also features the percentage of the final 277 articles each trend was identified. The “Potential Areas for Future Research” represent either complete gaps in the literature (i.e., not identified at all or 0%) or were aspects of research that were found far less frequently than the “Trends” but identified enough by team members to signify scholarly interest (typically appearing in roughly 5% or less of the literature). Put simply, these were aspects of the EC research that had received minimal attention in previous research, leaving ample room for future exploration and discovery. These “Potential Areas for Future Research” were agreed upon by the author team and are discussed in more depth below. The author team decided not to include potential areas for future research regarding *Conceptualization* to further promote and endorse a shared understanding of what is meant by “emotional contagion.” Regarding “Sample Characteristics,” the “Trends” listed pertain to age groups as the age of participants was the most commonly provided demographic characteristic of the sample. Race and ethnicity, gender, occupation, and other variables of interest were often not reported in the studies. Moreover, many studies simply offered the average age of participants, not necessarily a range. Therefore, what is presented in that column for the “Trends” stems from consensus among team members regarding the most frequently appearing age cohorts featured in the studies from their collective perspectives (i.e., no formal frequency analysis was conducted for this particular column).

**Table 1:** Most common trends and potential areas for future research.

Category	Primary area/topic of research	%	Conceptualization^a^	%	Triggers/stimuli	%	Measures	%	Sample characteristics
Most common trends	Adv. Psych-Discipline(s) Science	54	Hatfield et al. (1994)	33	Images/video of faces	38	Rating own/other’s emotional states (non-validated Likert scales, or single item questions)	28	Enrolled college students (ages 18–24)
	Workplace Culture/Org Leadership	12	Other “Hatfield et al.” citations (e.g., Hatfield et al., 1992, 1993, 2014, etc.)	20	Not triggered	32	Various emotion/empathy scales (e.g., ECWS, PANAS, BEES, IRI, etc.)	28	Adults (ages 25–50)
	Psychiatric/Autism/Brain Trauma/Substance abuse	11	No conceptualization offered	12	Real-time interactions/events	11	Emotional Contagion Scale (ECS) (Doherty, 1997)	26	Children (15 and younger)
	Marketing/Sales & Adv/Service Industry	11							
Potential areas for future research	Aging				Vignettes/Case Descriptions		Neuroimaging		Individuals from typically minoritized groups
	Clinical encounter				Vocalizations (non-visual)		Physiological Measures (e.g., skin conductance, HRV, saliva cortisol, etc.)		Older adults (65+)
	Education/school				Odorants		Wearable HR monitor		(purposeful) Race-discordant dyads/groups

a The %’s for the conceptualizations are relatively small because there were multiple versions of conceptualizations offered in the reports—23% of the reports utilized a citation for EC conceptualization that appeared only once or twice in the entire final review library. The conceptualizations presented in this table are those most frequently cited in the articles featured in the final review.

In turn, Table 1 orients the reader to the trends and gaps identified within EC research, and we provide a brief elaboration on these findings in the sub-sections that follow.

### Areas/topics of research

Most articles’ primary area/topic was classified as stemming from the psychology discipline and subdisciplines (e.g., social, cognitive, developmental, and neuropsychology). These studies were focused on advancing the psychological-oriented science of EC—extending and refining the research in this general arena regarding processes and mechanisms of EC (54%).

Additionally, team members identified work culture and organizational leadership as a frequently occurring research topic (12%). These articles typically featured examinations of superiors’ emotions on subordinates’ emotions, if/how EC (boss-worker) may relate to or affect productivity, and how workers’ self-reported degree of EC related to their workplace well-being and perspectives on the work environment and culture.

Another frequently identified area/topic of research was studies on EC levels among those living with autism spectrum-related diagnoses, psychiatric diagnoses, dementia, Alzheimer’s disease, mental illness, and/or brain trauma (11%) These articles explored to what extent (if any) do those living with substance use disorder, severe depression, Alzheimer’s disease, or severe brain injury connected with others emotionally, felt what others felt, and/or what particular areas of the brain were dormant, among other foci.

EC studies exploring interpersonal mechanisms and processes nested within the service or hospitality industry, marketing and advertising, and guest services in general (11%) tended to examine how the emotions of a service industry worker (e.g., waiter, receptionist, agent, etc.) influenced the emotional states of the “client,” and, in turn, how that potential connection influenced clients’ favorability with the service.

Additionally, articles exploring EC and social media (i.e., Facebook, Twitter) (5%) frequently explored how the emotionality nested within certain posts may/may not trigger observers/readers to create posts with similar emotionality. Other topics noted included: Computer Science, Art/Music, and Teamwork.

The research team identified aging (beyond Alzheimer’s-focused studies), clinical encounters (i.e., clinician-patient), and education/school (including teacher-student) as potential areas for future EC research, given their presence, albeit minimal, in the literature.

### Conceptualization

If a conceptualization was offered in the article (12% did not offer any conceptualization), the conceptualization of EC delivered by Hatfield et al. (1994), was the most frequently utilized conceptualization in the literature—either by including the full direct quotation and citation, citing a paraphrased version of it, or simply citing the authors in presenting the fundamentals of EC (33%).

Hatfield et al. (1994) define (primitive) emotional contagion as, “…the tendency to automatically mimic and synchronize facial expressions, vocalizations, postures, and movements with those of another person’s and, consequently, to converge emotionally” (p. 5).

Conceptualizations of EC featured in other “Hatfield et al.” studies (e.g., Hatfield et al., 1992, 1993, 2014, among others) were also frequently utilized in the articles (20%). Other notable conceptualizations featured in the articles were those provided by Doherty (1997) (5%) and Barsade (2002) (3%). Moreover, because studies on mimicry were included in this review, conceptualizations provided by Hess and colleagues (Hess and Fischer, 2013, 2014; Hess and Blairy, 2001; among others) appeared frequently as well (5%).

Doherty (1997) defines EC as, “…a multiply determined family of psychophysiological, cognitive, behavioral, and social phenomena in which eliciting stimuli arise from one individual, act upon one or more others, and produce emotional responses that are congruent (e.g., smiling response to smiles) or complimentary (e.g., withdrawal from a threatened blow) to the eliciting stimuli” (p. 134).

Hess and Fischer (2013) define emotional mimicry as, “…the imitation of the emotional expression of another person. By contrast we use the term emotional contagion to refer to the more generic process of ‘catching’ another’s emotion, without specifying the specific process that may underlie this outcome” (p. 142).^1^

As noted in Table 1, numerous articles in this review featured a conceptualization that only appeared once or twice in the entire final review.

### Triggers/stimuli

A majority of studies examined utilized videos or still images of facial expression of emotions (e.g., sadness, anger, happiness) to trigger EC, including those featured in Ekman and Friesen (1975), the ATR Facial Expression, and the MET-Core (38%). The author team identified real-time interactions/events as a common trend in the EC research—with researchers utilizing in-person interactions and encounters to trigger EC organically (11%). Short clips from TV shows and/or films were also found to be popular mechanisms to stimulate EC (6%). However, it should be noted that many studies actually did not “trigger” EC (32%), but rather assumed EC measured participants’ susceptibility to/with EC in various ways, typically through a self-report scale (discussed below).

The research team identified the use of Vignettes and Case Studies (i.e., written scenarios), vocalizations without accompanying visual stimuli, and odorants (e.g., sweat) as potential triggers/stimuli for future research.

#### Measures

In order to measure EC, we found that most studies relied on participants to rate their own and/or others’ emotional states, either pre and post stimuli, or simply post stimuli (28%). These “measures” were often non-validated Likert scales asking participants to report to what extent they felt a particular emotion or single item measures to assess if the participant felt a particular emotion. Similarly, 28% of articles utilized validated empathy and/or emotion-oriented scales such as the Positive and Negative Affect Schedule (PANAS), the Emotional Classification Method Scale (ECWS), or the Interpersonal Reactivity Index (IRI). OF these, the PANAS and the ECWS were the most common. Doherty’s (1997) Emotional Contagion Scale (ECS) was also utilized frequently (26%). Although featured in certain studies in tandem with other types of EC measurement procedures, this scale was often used in studies that did not “trigger” EC among dyads or groups but rather assessed participants’ self-reported levels of EC. Many articles featured more than one measurement tool.

It is important to note that although not as common a measurement as those reported above, EC was also measured by examining the activation (or non-activation) of participants’ facial muscles to assess emotion-specific responses (including mimicry) to an emotion-specific stimuli/trigger (e.g., did the participant engage in a “smile” after seeing a “happy” face and/or hear laughter). These types of measurement included electromyography (EMG), the facial expression analysis systems from iMotion, Ekman & Friesen’s FACS and OpenFace (14%). Similarly, 13% of articles engaged in some form of coding of participants’ facial expressions or body posture following exposure to some stimuli (e.g., facial expressions of emotion states). Many studies utilized more than one method of measurement (e.g., ECS *and* EMG).

The research team identified neuroimaging (e.g., assessing neural synchrony between brain networks integral for emotion during dyadic interactions, often referred to as “hyperscanning”), electrodermal response, and wearable heart rate monitors as potential mechanisms for measuring EC in future research.

#### Sample characteristics

As noted above, these aspects of the review are somewhat rudimentary in that no formal frequency analysis was conducted given the intricate nature of the sample or the lack of thorough reporting of sample characteristics. However, the author team identified what they felt were the most common samples utilized within the studies. These included college/university-aged students, “adults” ages 25–50 years, and children (ages 15 and younger).

Team members identified particular socio-demographic gaps in sampling techniques within the library of EC studies—notably, the general lack of non-white participants, older adults (65 years and older), and race-discordant dyads/groups—as potential areas for future research.

## Discussion

Whereas we present similar outcomes as other recent reviews of EC literature—such as the significant utilization of facial expressions to trigger EC and a need to further explore the use of neuroscience techniques to measure EC (e.g., fMRI)—our scoping review provides new focused insights in the common trends and potential next steps of EC research with human subjects. These include the prominent inclusion of Hatfield et al.’s (1994) conceptualization of EC, the substantial utilization of Doherty’s ECS to measure participants’ EC, a complete overreliance on self-report measures, and the (perhaps over-) reliance on samples of (typically majority white) college-age students in studies exploring processes of EC. These findings are not to suggest that EC research has gone stale or stagnant in any way—but rather to spotlight how and in what particular areas EC research with human participants needs to be expanded.

Interestingly, the Hatfield et al. (1994) citation references the authors’ book, *Emotional Contagion*, and yet the first full conceptualization provided in this book—the verbatim conceptualization featured in the majority of the articles within our data set—is actually a self-citation of their original conceptualization that appears in an edited volume (Hatfield et al., 1992). Potential mis-citing aside, it is also notable that many studies we reviewed (10%) did not feature *any* conceptualization of EC. Additionally, whereas it is useful that a strong majority of EC scholars are applying the same definition of EC in their research, given that EC is a contested aspect of the broader empathy experience, perhaps future work could explore particular nuances of the conceptualization through experimental research—dissecting the various aspects of the EC conceptualization in step-wise experimental design.

Differing conceptualizations of EC can have implications for how studies are designed and how their results are interpreted. If EC is conceptualized narrowly as an automatic, unconscious process of mimicry and emotional convergence, researchers may design studies that rely on nonverbal or sensory stimuli such as facial expressions, tone of voice, or bodily movements, typically delivered in controlled laboratory environments. As we see within this review, these studies tend to employ physiological or behavioral measures (e.g., facial EMG, heart rate) to detect mimicry-based responses. In contrast, broader conceptualizations that frame EC as involving cognitive appraisal or empathic understanding may lead to the use of more interpretive or narrative-based stimuli, such as emotionally charged scenarios or interpersonal dilemmas, or rely more heavily on self-report instruments or even neural imaging to assess internal emotional alignment. These slight shifts in conceptualize may also influence sample selection and study setting; automatic models may use general population samples in tightly controlled conditions, while broader models may focus on specific populations and naturalistic contexts. Moreover, interpretation of findings will vary with the conceptualization of EC as well: mechanistic definitions will allow for causal claims about emotion transfer, while broader ones support more contextual or relational interpretations. These differences shape both theoretical conclusions and practical applications of EC research, such as whether interventions in emotionally intense environments (like healthcare) focus on training nonverbal awareness or cultivating reflective emotional practices.

Given challenges cited in differing conceptualizations of empathy (Michalec and Hafferty, 2022), it is beneficial that the majority of emotional contagion research with human subjects utilizes the same conceptualization because it allows for consistency, comparability, and accumulation of knowledge across studies. When researchers define (and measure) emotional contagion in the same way, their findings can be more easily compared, replicated, and synthesized in meta-analyses, which strengthens the overall reliability and validity of the field. This shared framework also helps avoid confusion and misinterpretation of results, making it easier to build theory and apply findings in real-world settings like workplaces, schools, or healthcare.

It is not surprising that a majority of EC research could be codified as “mopping up” within the larger social-psychological discipline/field. Regarding human subjects, EC is a dynamic interpersonal phenomenon rooted in shared understandings and shared meanings of certain social cues and emotional representations, which fits entirely in the broad domain of social-psychology. Similarly, given the abundance of research, Barsade et al. (2018) recently presented a general review of EC literature as it relates to organizational life which they note includes: “…(1) team processes and outcomes, (2) leadership, (3) employee work attitudes, (4) decision-making, and (5) customer attitudes.” Therefore, it is not surprising that we found Workplace Culture/Organizational Leadership as a common trend regarding topic areas of EC research. Our review also highlights the prominence of scholarship exploring the processes and mechanisms of EC as experienced by individuals with psychiatric diagnoses, on the autism spectrum, as well as individuals who have suffered traumatic brain injuries. Given the recent attention to neurodiversity as well as practices and policies aimed at enhancing inclusion and diversity (broadly speaking), this particular arena of research will be extremely helpful in providing a better understanding of the social and psychological needs and experiences of individuals who have been previously excluded these studies.

Fruitful areas and topics for future EC research, as identified by the research team, include exploring EC processes and mechanisms (and outcomes) among older adults, and the role(s) and effects of EC within clinical settings, and within schools. Aging-related physiological, physical, and cognitive changes such as those related to muscle tension, facial structure, eyesight, hearing, speaking, and emotional processing, among others, influence how emotions are presented and received (Fölster et al., 2014; Gonçalves et al., 2018), and yet this particular population has been relatively neglected within EC research. Similarly, although previous research has explored how clinicians’ level of emotional sensitivity may be related to reported degrees of burnout (Costa and Moss, 2018; Jackson-Koku and Grime, 2019; Petitta et al., 2017; Le Blanc et al., 2001), there is scant empirical evidence regarding if or how EC positively or negatively affects clinicians’ well-being, patient satisfaction, or patient health outcomes. Additionally, we suggest that EC scholars delve further into how EC is experienced between teachers and students, teachers and teachers, and students and students, and whether aspects of these interactions play meaningful roles in fostering resilience, enhancing school culture and feelings of belongingness, and student and/or teacher retention. EC scholars could also explore how processes of the tenets of EC may affect group behavior in schools in emergency situations to improve safety protocols and practices.

Additionally, regarding methodological approaches, the research team identified neuroimaging, electrodermal response, and wearable heart rate monitors as potential mechanisms for measuring EC in future research, as this would allow researchers to examine the activation of emotion through additional physiological measures that are occurring in real time. Coupled with the self-reports, these activation measures may provide more accurate reports of EC as emotions may dissipate before a participant is asked to self-report their emotional state. Moreover, the research team identified the use of vignettes and case studies, vocalizations without accompanying visual stimuli, and odorants as potential triggers/stimuli for future research as such research could increase the usage of EC triggers in research and stimulate EC through novel, single mechanisms.

The abundant preference for Doherty’s ECS (1997), as well as a variety of miscellaneous scales and questions to measure participants’ (and/or interaction partners’) self-reported EC and/or emotional state, suggest a potential partiality to survey-based research. Although, as noted earlier, many studies in the review library employed some form of facial muscle assessment and/or featured multi-methods for measuring processes and mechanisms of EC, perhaps this substantial utilization of scales spotlights resource-related challenges for EC scholars. Given the longstanding understanding that caution is always warranted for any self-report measure (e.g., Nisbett and Wilson, 1977), but especially those based on ephemeral, contextually contingent constructs like emotion (e.g., appraisal-based theories of emotion; James, 1884; Lange, 1885; Schachter and Singer, 1962), it’s critical to highlight that much work is still needed in the EC literature to flesh out issues associated with internal and external validity of the measures most often used, and the temporal nature of EC in general (e.g., how rapidly it develops and devolves during dyadic interactions). Neuroscience-based measurement techniques, such as EEG and fMRI, and more physiological-based measurement techniques, like devices to assess heart rate, pupil dilation, and skin conductance (such as we suggest for future research), will be extremely helpful and necessary in isolating the parameters and boundary conditions of EC moving forward. While these approaches may require extensive resources including money, personnel (including specialists), and time, we echo Herrando and Constantinides (2021) that these types of neurological and physiological measurements can offer more precise, accurate, and effective data for future EC research.

Not discussed in previous EC literature reviews is the apparent overreliance on enrolled students for EC-based studies and the gap(s) such reliance has created within the research. It is essential that EC scholars expand their sample parameters to move beyond the antiquated practice of relying on convenience-based techniques to only include college-aged, typically white students (i.e., WEIRD students). Because of this focus, we currently know very little about EC processes and mechanisms in older adults and among non-white populations. If EC is an integral element of social connectivity, communication, and even community building, as consistently argued within previous research (de Waal and Preston, 2017; Barsade et al., 2018; Hazy and Boyatzis, 2015), then we must engage participants from various socio-demographic backgrounds.

Similarly, although there was *implicit* evidence of race discordant pairs within the studies included in the final review library, the discordance was not purposeful, nor the “feature” of the study. To explain further, with studies that presented a (somewhat) racially diverse sample (i.e., not *solely* white), the “interaction”/exchange between race-discordant pairs can be assumed; however, the discordance (and potential nuances in the EC processes stemming from this discordance) was not a stated aim of the study, nor was it explored in any depth when discussing findings or outcomes. The research team felt this was a sample-related feature that could be of significant interest to EC scholars, particularly those looking to expand on Allport’s Contact Hypothesis (Allport, 1954) as well as those interested in exploring how in- and out-group/network or social-proximity influences the “catching” of others’ emotional states (i.e., willingness and ability).

### Limitations

Despite the expansive nature of this scoping review, there are limitations to the study. The timeframe of the articles explored within this scoping review is substantial (10 years: 1992–2022), however, more recently published relevant articles were not included. Similarly, although all articles were diligently reviewed multiple times by multiple team members, it is possible that during the initial abstract review or the subsequent full-team review, certain articles were erroneously excluded that should have been included. Similarly, although unlikely, it is possible that errors were made in the categorization of articles and aspects of articles by *Primary Area/Topic of Research*, *Conceptualization, Triggers/Stimuli*, *Measurement*, and *Sample Characteristics*. Moreover, despite expansive search criteria via multiple databases, it is possible that EC studies were not included in the initial search process (as outlined in Figure 1). Finally, the “Potential Areas of Future Research” were identified and confirmed by members of this specific research team, which includes scholars in the areas of social neuroscience, sociology, medicine, health humanities, and health systems science. Team members from other discipline backgrounds or with other scholarly training may have identified other “areas” or directions for future EC research.

## Conclusion

This scoping review highlights significant strides and gaps in EC research, emphasizing the need for diversified approaches. Addressing conceptual, methodological, and demographic limitations is crucial for advancing EC research. Broader research scopes, inclusive populations, and innovative measurement techniques can deepen our understanding of EC, fostering healthier relationships and enhancing mental health outcomes. This comprehensive approach will enrich the theoretical framework of EC and inform practical interventions to improve social and emotional well-being.

## Data Availability

The original contributions presented in the study are included in the article/Supplementary material, further inquiries can be directed to the corresponding author.

## Footnotes

^1^Although Hess and Fischer (2013) define emotional mimicry in contrast to emotional contagion, because this particular work, as well as other works by Hess and colleagues (as noted above), were cited in a significant number of studies featured in this review on emotional mimicry *and* emotional contagion—we felt it is was essential to report here. However, this contrast in conceptualization does raise questions regarding the framework/foundation of previous EC studies that featured Hess and colleagues’ conceptualization of emotional mimicry.
