# Supplementary material for: A scoping review of emotional contagion research with human subjects: identifying common trends of previous research and potential areas for future research
Source: Front Psychol. 2025 May 30;16:1573375. doi: 10.3389/fpsyg.2025.1573375 (PMC12164912; doi:10.3389/fpsyg.2025.1573375)
Supplement: Supplementary file 1 [file Data_Sheet_1.docx]

**APPENDIX A: Detailed Description of Search Strategy**

**PsycInfo Februrary, 24 2022: 483 results**

(("emotional contagion" OR "emotional mimicry" OR "emotional sharing") AND (assess* OR instrument* OR measur* OR screen* OR questionnaire* OR survey* OR psychometric* OR scale* OR tool* OR evaluat* OR test* OR inventor* OR score* OR indicat* OR index* OR analyse* OR analyze OR electroencephalography OR electroencephalographies OR eeg OR signal* OR hrv OR "blood oxygen level dependent" OR sympathetic* OR "heart rate")) NOT (POP(animal) NOT POP(human))

Limited by:Peer reviewed Date: After January 01 1992

Language:English

**Scopus February 24, 2022: 620 results**

TITLE-ABS-KEY((assess* OR instrument* OR measur* OR screen* OR questionnaire* OR survey* OR psychometric* OR scale* OR tool* OR evaluat* OR test* OR inventor* OR score* OR indicat* OR index* OR analyse* OR analyze OR electroencephalography OR electroencephalographies OR eeg OR signal* OR hrv OR "blood oxygen level dependent" OR sympathetic* OR "heart rate") AND ("emotional contagion" OR "emotional sharing" OR "emotional mimicry")) AND PUBYEAR > 1991 AND NOT ((INDEXTERMS(animals OR animal)) AND NOT (INDEXTERMS(humans OR human))) AND ( LIMIT-TO ( DOCTYPE , "ar" ) OR LIMIT-TO ( DOCTYPE , "re" ) ) AND ( LIMIT-TO ( LANGUAGE,"English" ) )

**Biological Sciences February, 25 2022: 198 results**

(noft("emotional contagion" OR "emotional mimicry" OR "emotional sharing") AND (assess* OR instrument* OR measur* OR screen* OR questionnaire* OR survey* OR psychometric* OR scale* OR tool* OR evaluat* OR test* OR inventor* OR score* OR indicat* OR index* OR analyse* OR analyze OR electroencephalography OR electroencephalographies OR eeg OR signal* OR hrv OR "blood oxygen level dependent" OR sympathetic* OR "heart rate")) NOT ((Animal) NOT (human OR men OR women))

Filters Applied: Scholarly Journals 1992-01-01 - 2022 English NOT MEDLINE® Peer Reviewed

**PubMed March 3, 2022: 288 results**

("emotional contagion" OR "emotional mimicry" OR "emotional sharing") AND (assessment OR instrument OR measurement OR score OR inventory OR screening OR questionnaire OR survey OR psychometric OR scale OR tool OR evaluation OR test OR indicator OR index OR analysis OR analyze OR electroencephalography OR EEG OR signal OR heart rate OR HRV OR "blood oxygen level dependent" OR sympathetic) NOT ("animals"[mh] NOT "humans"[mh])

Filters: from 1992 – 2022, English

**Google Scholar March 5, 2022: First 50 results**

("emotional contagion" OR "emotional mimicry" OR "emotional sharing") (assessment OR instrument OR measurement OR measure OR questionnaire OR survey OR tool OR test OR measurement OR analysis OR indicator OR screening OR inventory OR index) -animal
