# Supplementary material for: A scoping review of emotional contagion research with human subjects: identifying common trends of previous research and potential areas for future research
Source: Front Psychol. 2025 May 30;16:1573375. doi: 10.3389/fpsyg.2025.1573375 (PMC12164912; doi:10.3389/fpsyg.2025.1573375)
Supplement: Supplementary file 2 [file Data_Sheet_2.docx]

**APPENDIX B: Full Scoping Review Reference List (277 Articles)**

Achaibou, A., Pourtois, G., Schwartz, S., & Vuilleumier, P. (2008). Simultaneous recording of EEG and facial muscle reactions during spontaneous emotional mimicry. *Neuropsychologia*, 46(4), 1104-1113. <https://doi.org/10.1016/j.neuropsychologia.2007.10.019>

Achour-Benallegue, A., Amarantini, D., Paubel, P.-V., Pelletier, J., & Kaminski, G. (2021). Facial reactions to face representations in art: An electromyography study. *Psychology of Aesthetics, Creativity, and the Arts,* 17(6), 793-805. Advance online publication. <https://doi.org/10.1037/aca0000423>

Ahmad, F., & Guzmán, F. (2021). Negative online reviews, brand equity and emotional contagion. *European Journal of Marketing*, 55(11), 2825-2870. <https://doi.org/10.1108/EJM-10-2019-0820>

Akkermans, J., Schapiro, R., Müllensiefen, D., Jakubowski, K., Shanahan, D., Baker, D., Busch, V., Lothwesen, K., Elvers, P., Fischinger, T., Schlemmer, K., & Frieler, K. (2019). Decoding emotions in expressive music performances: A multi-lab replication and extension study. *Cognition and Emotion*, 33(6), 1099-1118. <https://doi.org/10.1080/02699931.2018.1541312>

Alluri, V., Mittal, A., Sc, A., Vuoskoski, J. K., & Saarikallio, S. (2022). Maladaptive music listening strategies are modulated by individual traits. *Psychology of Music,* 50(6), 1779–1800. <https://doi.org/10.1177/03057356211065061>

Almaliah-Rauscher, S., Ettinger, N., Levi-Belz, Y., & Gvion, Y. (2020). “Will you treat me? I'm suicidal!” the effect of patient gender, suicidal severity, and therapist characteristics on the therapist's likelihood to treat a hypothetical suicidal patient. *Clinical Psychology & Psychotherapy*, 27(3), 278-287. <https://doi.org/10.1002/cpp.2426>

Amat, A., Zapata, C., Alexakos, K., Pride, L. D., Paylor-Smith, C., & Hernandez, M. (2016). Incorporating oximeter analyses to investigate synchronies in heart rate while teaching and learning about race. *Cultural Studies of Science Education,* 11(3), 785-801. <https://doi.org/10.1007/s11422-016-9767-z>

Anitha, E. G. N., & Suganthi, L. (2022). How organizational citizenship behavior is promoted among nurses: A moderated mediation model. *Work*, 71(1), 263-274. <https://doi.org/10.3233/WOR-210329>

Arnold, A. J., & Winkielman, P. (2021). Smile (but only deliberately) though your heart is aching: Loneliness is associated with impaired spontaneous smile mimicry. *Social Neuroscience*, 16(1), 26-38. <https://doi.org/10.1080/17470919.2020.1809516>

Bae, M. (2019). Influences of identified victim images on processing fluency. J*ournal of Nonprofit & Public Sector Marketing*, 31(3), 249-273. <https://doi.org/10.1080/10495142.2018.1526740>

Bakker, A. B., & Schaufeli, W. B. (2000). Burnout contagion processes among teachers. *Journal of Applied Social Psychology,* 30(11), 2289-2308. <https://doi.org/10.1111/j.1559-1816.2000.tb02437.x>

Bakker, A. B., Schaufeli, W. B., Sixma, H. J., & Bosveld, W. (2001). Burnout contagion among general practitioners. *Journal of Social and Clinical Psychology,* 20(1), 82-98. <https://doi.org/10.1521/jscp.20.1.82.22251>

Balconi, M., & Bortolotti, A. (2012). Empathy in cooperative versus non-cooperative situations: The contribution of self-report measures and autonomic responses. *Applied Psychophysiology and Biofeedback,* 37(3), 161-169. <https://doi.org/10.1007/s10484-012-9188-z>

Baral, R., & Sampath, P. (2019). Exploring the moderating effect of susceptibility to emotional contagion in the crossover of work–family conflict in supervisor–subordinate dyads in India. *Personnel Review,* 48(5), 1336-1356. <https://doi.org/10.1108/PR-05-2017-0139>

Barger, P. B., & Grandey, A. A. (2006). Service with a smile and encounter satisfaction: Emotional contagion and appraisal mechanisms. *Academy of Management Journal,* 49(6), 1229-1238. <https://doi.org/10.5465/AMJ.2006.23478695>

Barsade, S. G. (2002). The ripple effects: Emotional contagion and its influence on group behavior. *Administrative Science Quarterly,* 47(4), 644-675. <https://doi.org/10.2307/3094912>

Bayot, M., Vermeulen, N., Kever, A., & Mikolajczak, M. (2020). Mindfulness and empathy: Differential effects of explicit and implicit Buddhist teachings. *Mindfulness*, 11(1), 5-17. <https://doi.org/10.1007/s12671-018-0966-4>

Belli, S., & Alonso, C. V. (2021). COVID-19 pandemic and emotional contagion. *DigitHVM Revista Digital d’Humanitats*, (27), 1-9. <https://doi.org/10.7238/D.V0I27.374153>

Berg, H., Söderlund, M., & Lindström, A. (2015). Spreading joy: Examining the effects of smiling models on consumer joy and attitudes. *Journal of Consumer Marketing*, 32(6), 459-469. <https://doi.org/10.1108/JCM-03-2015-1356>

Berger, P., Bitsch, F., Jakobi, B., Nagels, A., Straube, B., & Falkenberg, I. (2019). Cognitive and emotional empathy in patients with schizophrenia spectrum disorders: A replication and extension study. *Psychiatry Research*, 276, 56-59. <https://doi.org/10.1016/j.psychres.2019.04.015>

Bhullar, N. (2012a). Relationship between mood & susceptibility to emotional contagion: Is positive mood more contagious? *North American Journal of Psychology*, 14(3), 517-530.

Bhullar, N. (2012b). Self-ratings of love and fear on Emotional Contagion Scale depend on the environmental context of rating. *Current Research in Social Psychology*, 19(1), Article 9.

Borawski, D., Wajs, T., Sojka, K., & Misztal, U. (2021). Interrelations between attachment styles, emotional contagion and loneliness. *Journal of Family Issues*, 42(9), 2064-2082. <https://doi.org/10.1177/0192513X20966013>

Cannon, P. R., Hayes, A. E., & Tipper, S. P. (2009). An electromyographic investigation of the impact of task relevance on facial mimicry. *Cognition and Emotion*, 23(5), 918-929. <https://doi.org/10.1080/02699930802234864>

Cardeña, E., Terhune, D. B., Lööf, A., & Buratti, S. (2009). Hypnotic experience is related to emotional contagion. *International Journal of Clinical and Experimental Hypnosis*, 57(1), 33-46. <https://doi.org/10.1080/00207140802463500>

Chabin, T., Tio, G., Comte, A., Joucla, C., Gabriel, D., & Pazart, L. (2020). The relevance of a conductor competition for the study of emotional synchronization within and between groups in a natural musical setting. *Frontiers in Psychology*, 10, Article 11. <http://dx.doi.org/10.3389/fpsyg.2019.02954>

Chancellor, J., Layous, K., Margolis, S., & Lyubomirsky, S. (2017). Clustering by well-being in workplace social networks: Homophily and social contagion. *Emotion*, 17(8), 1166-1180. <https://doi.org/10.1037/emo0000311>

Chatel-Goldman, J., Congedo, M., Jutten, C., & Schwartz, J.-L. (2014). Touch increases autonomic coupling between romantic partners. *Frontiers in Behavioral Neuroscience*, 8, Article 95. <https://doi.org/10.3389/fnbeh.2014.00095>

Chen, W., McDonald, S., Wearne, T., & Grisham, J. (2021). Investigating associations between hoarding symptoms and affective and cognitive empathy. *British Journal of Clinical Psychology*, 60(2), 177-193. <http://dx.doi.org/10.1111/bjc.12280>

Cheng, Y.-N., Yen, C.-L., & Chen, L. H. (2012). Transformational leadership and job involvement: The moderation of emotional contagion. *Military Psychology*, 24(4), 382-396. <http://dx.doi.org/10.1080/08995605.2012.695261>

Cherulnik, P. D., Donley, K. A., Wiewel, T. S. R., & Miller, S. R. (2001). Charisma is contagious: The effect of leaders' charisma on observers' affect. *Journal of Applied Social Psychology*, 31(10), 2149-2159. <https://doi.org/10.1111/j.1559-1816.2001.tb00167.x>

Choi, S., & Kim, E.-M. (2020). Between Instagram browsing and subjective well-being: Social comparison or emotional contagion? *Media Psychology*, 24(6) 866-890. <https://doi.org/10.1080/15213269.2020.1824120>

Chu, K. H., Baker, M. A., & Murrmann, S. K. (2012). When we are onstage, we smile: The effects of emotional labor on employee work outcomes. I*nternational Journal of Hospitality Management*, 31(3), 906-915. <https://doi.org/10.1016/j.ijhm.2011.10.009>

Cohen, E. L., & Bowman, N. D., & Lancaster, A. L. (2016). R U with Some1? Using text message experience sampling to examine television coviewing as a moderator of emotional contagion effects on enjoyment. *Mass Communication & Society*, 19(2), 149-172. <https://doi.org/10.1080/15205436.2015.1071400>

Cohen, E. L., & Lancaster, A. L. (2014). Individual differences in in-person and social media television coviewing: The role of emotional contagion, need to belong, and coviewing orientation. *Cyberpsychology, Behavior, and Social Networking*, 17(8), 512-518. <https://doi.org/10.1089/cyber.2013.0484>

Cotterill, S. T., Clarkson, B. G., & Fransen, K. (2020). Gender differences in the perceived impact that athlete leaders have on team member emotional states. J*ournal of Sports Sciences*, 38(10), 1181-1185. <https://doi.org/10.1080/02640414.2020.1745460>

Coviello, L., Sohn, Y., Kramer, A. D. I., Marlow, C., Franceschetti, M., Christakis, N. A., & Fowler, J. H. (2014). Detecting emotional contagion in massive social networks. *PLoS ONE*, 9(3), Article e90315. <https://doi.org/10.1371/journal.pone.0090315>

Crocamo, C., Viviani, M., Famiglini, L., Bartoli, F., Pasi, G., & Carrà, G. (2021). Surveilling COVID-19 emotional contagion on Twitter by sentiment analysis. *European Psychiatry: The Journal of the Association of European Psychiatrists*, 64(1), Article e17. <https://doi.org/10.1192/j.eurpsy.2021.3>

Cui, X. (2018). Emotional contagion or symbolic cognition? A social identity perspective on media events. *Journal of Broadcasting & Electronic Media*, 62(1), 91-108. <http://dx.doi.org/10.1080/08838151.2017.1402906>

Cummins, R. G., & Cui, B. (2014). Reconceptualizing address in television programming: The effect of address and affective empathy on viewer experience of parasocial interaction. *Journal of Communication*, 64(4), 723-742. <https://doi.org/10.1111/jcom.12076>

Czarna, A. Z., Wróbel, M., Dufner, M., & Zeigler-Hill, V. (2015). Narcissism and emotional contagion: Do narcissists “catch” the emotions of others? *Social Psychological and Personality Science*, 6(3), 318-324. <https://doi.org/10.1177/1948550614559652>

Dallimore, K. S., Sparks, B. A., & Butcher, K. (2007). The influence of angry customer outbursts on service providers' facial displays and affective states. *Journal of Service Research*, 10(1), 78-92. <https://doi.org/10.1177/1094670507304694>

de Groot, J. H. B., Semin, G. R., & Smeets, M. A. M. (2014). I can see, hear, and smell your fear: Comparing olfactory and audiovisual media in fear communication. *Journal of Experimental Psychology: General*, 143(2), 825-834. <https://doi.org/10.1037/a0033731>

de Groot, J. H. B., Smeets, M. A. M., Kaldewaij, A., Duijndam, M. J. A., & Semin, G. R. (2012). Chemosignals communicate human emotions. *Psychological Science*, 23(11), 1417-1424. <https://doi.org/10.1177/0956797612445317>

de Sousa, A., McDonald, S., & Rushby, J. (2012). Changes in emotional empathy, affective responsivity, and behavior following severe traumatic brain injury. *Journal of Clinical and Experimental Neuropsychology,* 34(6), 606-623. <https://doi.org/10.1080/13803395.2012.667067>

Deng, H., & Hu, P. (2018). Matching your face or appraising the situation: Two paths to emotional contagion. *Frontiers in Psychology*, 8, Article 2278. <https://doi.org/10.3389/fpsyg.2017.02278>

Dethier, M., & Blairy, S. (2012). Capacity for cognitive and emotional empathy in alcohol-dependent patients. *Psychology of Addictive Behaviors,* 26(3), 371-383. <https://doi.org/10.1037/a0028673>

Dezecache, G., Conty, L., Chadwick, M., Philip, L., Soussignan, R., Sperber, D., & Grèzes, J. (2013). Evidence for unintentional emotional contagion beyond dyads. *PLoS ONE,* 8(6), Article e67371. <https://doi.org/10.1371/journal.pone.0067371>

Dijk, C., Fischer, A. H., Morina, N., Eeuwijk, C., & Kleef, G. A. (2018). Effects of social anxiety on emotional mimicry and contagion: Feeling negative, but smiling politely. *Journal of Nonverbal Behavior,* 42(1), 81-99. <https://doi.org/10.1007/s10919-017-0266-z>

Dimberg, U., & Thunberg, M. (2012). Empathy, emotional contagion, and rapid facial reactions to angry and happy facial expressions. *PsyCh Journal,* 1(2), 118-127. <http://dx.doi.org/10.1002/pchj.4>

Doherty, R. W. (1997). The emotional contagion scale: A measure of individual differences. *Journal of Nonverbal Behavior,* 21(2), 131-154. <https://doi.org/10.1023/A:1024956003661>

Doherty, R. W. (1998). Emotional contagion and social judgment. *Motivation and Emotion,* 22(3), 187-209. <https://doi.org/10.1023/A:1022368805803>

Doherty, R. W., Orimoto, L., Singelis, T. M., Hatfield, E., & Hebb, J. (1995). Emotional contagion: Gender and occupational differences. *Psychology of Women Quarterly,* 19(3), 355-371. <https://doi.org/10.1111/j.1471-6402.1995.tb00080.x>

Du, J., Fan, X., & Feng, T. (2011). Multiple emotional contagions in service encounters. *Journal of the Academy of Marketing Science,* 39(3), 449-466. <https://doi.org/10.1007/s11747-010-0210-9>

Du, J., Fan, X., & Feng, T. (2014). Group emotional contagion and complaint intentions in group service failure: The role of group size and group familiarity. *Journal of Service Research,* 17(3), 326-338. <https://doi.org/10.1177/1094670513519290>

Eerola, T., Vuoskoski, J. K., & Kautiainen, H. (2016). Being moved by unfamiliar sad music is associated with high empathy. *Frontiers in Psychology,* 7, Article 12. <https://doi.org/10.3389/fpsyg.2016.01176>

Efimov, K., Ntoumanis, I., Kuskova, O., Kadieva, D., Panidi, K., Kosonogov, V., Kazanina, N., Shestakova, A., Klucharev, V., & Jääskeläinen, I. P. (2022). Impact of induced moods, sensation seeking, and emotional contagion on economic decisions under risk. *Frontiers in Psychology,* 12, Article 796016. <https://doi.org/10.3389/fpsyg.2021.796016>

Egermann, H., & McAdams, S. (2013). Empathy and emotional contagion as a link between recognized and felt emotions in music listening. *Music Perception,* 31(2), 139-156. <https://doi.org/10.1525/mp.2013.31.2.139>

Escobar-Viera, C., Shensa, A., Hamm, M., Melcher, E. M., Rzewnicki, D. I., Egan, J. E., Sidani, J. E., & Primack, B. A. (2020). “I don’t feel like the odd one”: Utilizing content analysis to compare the effects of social media use on well-being among sexual minority and nonminority US young adults. *American Journal of Health Promotion,* 34(3), 285-293. <https://doi.org/10.1177/0890117119885517>

Fairbairn, C. E., Sayette, M. A., Aalen, O. O., & Frigessi, A. (2015). Alcohol and emotional contagion: An examination of the spreading of smiles in male and female drinking groups. *Clinical Psychological Science,* 3(5), 686-701. <https://doi.org/10.1177/2167702614548892>

Falkenberg, I., Bartels, M., & Wild, B. (2008). Keep smiling!: Facial reactions to emotional stimuli and their relationship to emotional contagion in patients with schizophrenia. *European Archives of Psychiatry and Clinical Neuroscience,* 258(4), 245-253. <https://doi.org/10.1007/s00406-007-0792-5>

Fan, Y.-T., Hsu, Y.-Y., & Cheng, Y. (2013). Sex matters: *n*-back modulates emotional mismatch negativity. *NeuroReport: For Rapid Communication of Neuroscience Research*, 24(9), 457-463. <http://dx.doi.org/10.1097/WNR.0b013e32836169b9>

Fehrenbacher, D. D. (2017). Affect infusion and detection through faces in computer-mediated knowledge-sharing decisions. *Journal of the Association for Information Systems,* 18(10), 703-726. <https://doi.org/10.17705/1jais.00470>

Feldman, D. B., & Kaal, K. J. (2007). Vicarious trauma and assumptive worldview: Beliefs about the world in acquaintances of trauma victims. *Traumatology,* 13(3), 21-31. <https://doi.org/10.1177/1534765607305437>

Ferrara, E., & Yang, Z. (2015). Measuring emotional contagion in social media. *PLoS ONE*, 10(11), Article e0142390. <https://doi.org/10.1371/journal.pone.0142390>

Filipowicz, A., Barsade, S., & Melwani, S. (2011). Understanding emotional transitions: The interpersonal consequences of changing emotions in negotiations. *Journal of Personality and Social Psychology,* 101(3), 541-556. <https://doi.org/10.1037/a0023545>

Fischer, A. H., Becker, D., & Veenstra, L. (2012). Emotional mimicry in social context: The case of disgust and pride. *Frontiers in Psychology,* 3, Article 475. <https://doi.org/10.3389/fpsyg.2012.00475>

Foroni, F., & Semin, G. R. (2011). When does mimicry affect evaluative judgment? *Emotion*, 11(3), 687-690. <https://doi.org/10.1037/a0023163>

Fox, A. K., Deitz, G. D., Royne, M. B., & Fox, J. D. (2018). The face of contagion: Consumer response to service failure depiction in online reviews. *European Journal of Marketing,* 52(1/2), 39-65. <https://doi.org/10.1108/EJM-12-2016-0887>

Freemantle, A. W. J., Stafford, L. D., Wagstaff, C. R. D., & Akehurst, L. (2021). The relationship between olfactory function and emotional contagion. *Chemosensory Perception.* 15(2), 49-59. <https://doi.org/10.1007/s12078-021-09293-4>

Frenzel, A. C., Becker-Kurz, B., Pekrun, R., Goetz, T., & Lüdtke, O. (2018). Emotion transmission in the classroom revisited: A reciprocal effects model of teacher and student enjoyment. *Journal of Educational Psychology,* 110(5), 628-639. <https://doi.org/10.1037/edu0000228>

Frisby, B. N. (2019). The influence of emotional contagion on student perceptions of instructor rapport, emotional support, emotion work, valence, and cognitive learning. *Communication Studies,* 70(4), 492-506. <https://doi.org/10.1080/10510974.2019.1622584>

Fujimura, T., & Okanoya, K. (2016). Untrustworthiness inhibits congruent facial reactions to happy faces. *Biological Psychology,* 121(Part A), 30-38. <http://dx.doi.org/10.1016/j.biopsycho.2016.09.005>

Gabriel, O. W., & Masch, L. (2017). Displays of emotion and citizen support for Merkel and Gysi: How emotional contagion affects evaluations of leadership. *Politics and the Life Sciences,* 36(2), 80-103. <https://doi.org/10.1017/pls.2017.15>

Garrido, S., & Macritchie, J. (2020). Audience engagement with community music performances: Emotional contagion in audiences of a ‘pro-am’ orchestra in suburban Sydney. *Musicae Scientiae,* 24(2), 155-167. <https://doi.org/10.1177/1029864918783027>

Geangu, E., Benga, O., Stahl, D., & Striano, T. (2010). Contagious crying beyond the first days of life. *Infant Behavior and Development,* 33(3), 279-288. <https://doi.org/10.1016/j.infbeh.2010.03.004>

Gerbaudo, P. (2016). Rousing the Facebook crowd: Digital enthusiasm and emotional contagion in the 2011 protests in Egypt and Spain. *International Journal of Communication*, 10(1), 254-273.

Gerger, G., Pelowski, M., & Leder, H. (2018). Empathy, Einfühlung, and aesthetic experience: The effect of emotion contagion on appreciation of representational and abstract art using fEMG and SCR. *Cognitive Processing,* 19(2), 147-165. <https://doi.org/10.1007/s10339-017-0800-2>

Gilal, F. G., Channa, N. A., Gilal, N. G., Gilal, R. G., & Shah, S. M. M. (2019). Association between a teacher’s work passion and a student’s work passion: A moderated mediation model. *Psychology Research and Behavior Management,* 12, 611-623. <https://doi.org/10.2147/PRBM.S212004>

Gilal, F. G., Gilal, N. G., Channa, N. A., Gilal, R. G., & Tunio, M. N. (2020). Towards an integrated model for the transference of environmental responsibility. *Business Strategy and the Environment,* 29(6), 2614-2623. <https://doi.org/10.1002/bse.2524>

Gilal, F. G., Zhang, J., Gilal, N. G., & Gilal, R. G. (2018). Association between a parent’s brand passion and a child’s brand passion: A moderated moderated-mediation model. *Psychology Research and Behavior Management,* 11, 91-102. <http://doi.org/10.2147/PRBM.S212004>

Goodman, C. R., & Shippy, R. A. (2002). Is it contagious? Affect similarity among spouses. *Aging & Mental Health,* 6(3), 266-274. <https://doi.org/10.1080/13607860220142431>

Gountas, S., Gountas, J., Soutar, G., & Mavondo, F. (2014). Delivering good service: Personal resources, job satisfaction and nurses’ ‘customer’ (patient) orientation. *Journal of Advanced Nursing,* 70(7), 1553-1563. <https://doi.org/10.1111/jan.12308>

Gouveia, V. V., Gouveia, R. S. V., Guerra, V. M., Santos, W. S., & de Medeiros, E. D. (2007). Measuring emotional contagion: Adaptation of the Doherty scale. *Revista de Psicologia Social,* 22(2), 99-111. <https://doi.org/10.1174/021347407780705401>

Grynberg, D., Maurage, P., & Nandrino, J.-L. (2017). Preserved affective sharing but impaired decoding of contextual complex emotions in alcohol dependence. *Alcoholism: Clinical and Experimental Research,* 41(4), 779-785. <https://doi.org/10.1111/acer.13330>

Guadagno, R. E., Rempala, D. M., Murphy, S., & Okdie, B. M. (2013). What makes a video go viral? An analysis of emotional contagion and Internet memes. *Computers in Human Behavior,* 29(6), 2312-2319. <https://doi.org/10.1016/j.chb.2013.04.016>

Gump, B. B., & Kulik, J. A. (1997). Stress, affiliation, and emotional contagion. *Journal of Personality and Social Psychology,* 72(3), 305-319. <https://doi.org/10.1037/0022-3514.72.2.305>

Guo, X., Zheng, L., Wang, H., Zhu, L., Li, J., Wang, Q., Dienes, Z., & Yang, Z. (2013). Exposure to violence reduces empathetic responses to other’s pain. *Brain and Cognition,* 82(2), 187-191. <http://dx.doi.org/10.1016/j.bandc.2013.04.005>

Han, M., Lee, S. E., & Lee, P. A. (2012). Burnout among entering MSW students: Exploring the role of personal attributes. *Journal of Social Work Education,* 48(3), 439-457. <https://doi.org/10.5175/JSWE.2011.201000053>

Hasford, J., Hardesty, D. M., & Kidwell, B. (2015). More than a feeling: Emotional contagion effects in persuasive communication. *Journal of Marketing Research,* 52(6), 836-847. <https://doi.org/10.1509/jmr.13.0081>

Hashim, J., Wok, S., & Ghazali, R. (2008). Organisational behaviour associated with emotional contagion among direct selling members. *Direct Marketing: An International Journal,* 2(3), 144-158. <https://doi.org/10.1108/17505930810899302>

Helt, M. S., Fein, D. A., & Vargas, J. E. (2020). Emotional contagion in children with autism spectrum disorder varies with stimulus familiarity and task instructions. *Development and Psychopathology,* 32(1), 383-393. <https://doi.org/10.1017/S0954579419000154>

Hennig-Thurau, T., Groth, M., Paul, M., & Gremler, D. D. (2006). Are All Smiles Created Equal? How Emotional Contagion and Emotional Labor Affect Service Relationships. *Journal of Marketing,* 70(3), 58-73. <http://dx.doi.org/10.1509/jmkg.70.3.58>

Hervé, M. J., Grandjean, H., Visier, J. P., & Maury, M. (2009). Triadic nonverbal communication in mother-infant consultations: Two contrasted cases. *Infant Mental Health Journal,* 30(3), 245-264. <https://doi.org/10.1002/imhj.20213>

Hess, U., & Blairy, S. (2001). Facial mimicry and emotional contagion to dynamic emotional facial expressions and their influence on decoding accuracy. *International Journal of Psychophysiology,* 40(2), 129-141. <http://dx.doi.org/10.1016/S0167-8760(00)00161-6>

Hess, U., Blaison, C., & Dandeneau, S. (2017). The impact of rewards on empathic accuracy and emotional mimicry. *Motivation and Emotion,* 41(1), 107-112. <http://dx.doi.org/10.1007/s11031-016-9590-6>

Hietanen, J. K., Surakka, V., & Linnankoski, I. (1998). Facial electromyographic responses to vocal affect expressions. *Psychophysiology,* 35(5), 530-536. <https://doi.org/10.1017/S0048577298970445>

Hill, E. M., Griffiths, F. E., & House, T. (2015). Spreading of healthy mood in adolescent social networks. *Proceedings of the Royal Society B: Biological Sciences,* 282(1813), Article 20151180. <https://doi.org/10.1098/rspb.2015.1180>

Holman, D., & Niven, K. (2019). Does interpersonal affect regulation influence others’ task performance? The mediating role of positive mood. *European Journal of Work and Organizational Psychology,* 28(6), 820-830. <https://doi.org/10.1080/1359432X.2019.1666105>

Howard, D. J., & Gengler, C. (2001). Emotional contagion effects on product attitudes. *Journal of Consumer Research,* 28(2), 189-201. <https://doi.org/10.1086/322897>

Hsee, C. K., Hatfield, E., & Chemtob, C. (1992). Assessments of the emotional states of others: Conscious judgments versus emotional contagion. *Journal of Social and Clinical Psychology,* 11(2), 119-128. <https://doi.org/10.1521/jscp.1992.11.2.119>

Hubner, S., Baum, M., & Frese, M. (2020). Contagion of entrepreneurial passion: Effects on employee outcomes. *Entrepreneurship Theory & Practice,* 44(6), 1112-1140. <https://doi.org/10.1177/1042258719883995>

Hühnel, I., Kuszynski, J., Asendorpf, J. B., & Hess, U. (2018). Emotional mimicry of older adults’ expressions: Effects of partial inclusion in a Cyberball paradigm. *Cognition and Emotion,* 32(1), 92-101. <https://doi.org/10.1080/02699931.2017.1284046>

Ilies, R., Wagner, D. T., & Morgeson, F. P. (2007). Explaining affective linkages in teams: Individual differences in susceptibility to contagion and individualism-collectivism. *Journal of Applied Psychology,* 92(4), 1140-1148. <https://doi.org/10.1037/0021-9010.92.4.1140>

Järvelä, S., Kätsyri, J., Ravaja, N., Chanel, G., & Henttonen, P. (2016). Intragroup Emotions: Physiological Linkage and Social Presence. *Frontiers in Psychology,* 7, Article 105. <https://doi.org/10.3389/fpsyg.2016.00105>

Jensen, M. (2019). Emotional contagion and group affective tone in the student cohort model. *American Journal of Occupational Therapy,* 73(4) Article 7304205120p1. <https://doi.org/10.5014/ajot.2019.032292>.

Jia, M., & Cheng, J. (2021). Emotional experiences in the workplace: Biological sex, supervisor nonverbal behaviors, and subordinate susceptibility to emotional contagion. *Psychological Reports,* 124(4), 1687-1714. <https://doi.org/10.1177/0033294120940552>

Jin, Z., Zhao, K. B., Xia, Y. Y., Chen, R. J., Yu, H., Tamunang Tamutana, T., Yuan, Z., Shi, Y. M., Adamseged, H. Y., Kogay, M., & Park, G. Y. (2020). Relationship between psychological responses and the appraisal of risk communication during the early phase of the COVID-19 pandemic: A two-wave study of community residents in China. *Frontiers in Public Health,* 8, Article 550220. <http://dx.doi.org/10.3389/fpubh.2020.550220>

Johnson, S. K. (2008). I second that emotion: Effects of emotional contagion and affect at work on leader and follower outcomes. *The Leadership Quarterly,* 19(1), 1-19. <https://doi.org/10.1016/j.leaqua.2007.12.001>

Jung, H. S., & Yoon, H. H. (2019). Emotional contagion and collective commitment among leaders and team members in deluxe hotel. *Service Business,* 13(4), 737-754. <https://doi.org/10.1007/s11628-019-00403-7>

Juslin, P. N., Harmat, L., & Laukka, P. (2018). The wisdom of the body: Listeners' autonomic arousal distinguishes between spontaneous and posed vocal emotions. *Scandinavian Journal of Psychology,* 59(2), 105-112. <https://doi.org/10.1111/sjop.12429>

Juszkiewicz, A., Lachowicz-Tabaczek, K., & Wróbel, M. (2020). Self-esteem, gender, and emotional contagion: What predicts people’s proneness to “catch” the feelings of others? *Personality and Individual Differences,* 157, Article 109803. <https://doi.org/10.1016/j.paid.2019.109803>

Kang, J., Derva, D., Kwon, D.-Y., & Wallraven, C. (2019). Voluntary and spontaneous facial mimicry toward other’s emotional expression in patients with Parkinson’s disease. *PLoS ONE,* 14(4), Article e0214957. <https://doi.org/10.1371/journal.pone.0214957>

Kastendieck, T., Mauersberger, H., Blaison, C., Ghalib, J., & Hess, U. (2021). Laughing at funerals and frowning at weddings: Top-down influences of context-driven social judgments on emotional mimicry. *Acta Psychologica,* 212, Article 103195. <http://dx.doi.org/10.1016/j.actpsy.2020.103195>

Kastendieck, T., Zillmer, S., & Hess, U. (2022). (Un)mask yourself! Effects of face masks on facial mimicry and emotion perception during the COVID-19 pandemic. *Cognition & Emotion,* 36(1), 59-69. <http://dx.doi.org/10.1080/02699931.2021.1950639>

Kelly, J. R., Iannone, N. E., & McCarty, M. K. (2016). Emotional contagion of anger is automatic: An evolutionary explanation. *British Journal of Social Psychology,* 55(1), 182-191. <https://doi.org/10.1111/bjso.12134>

Kevrekidis, P., Skapinakis, P., Damigos, D., & Mavreas, V. (2008). Adaptation of the Emotional Contagion Scale (ECS) and gender differences within the Greek cultural context. *Annals of General Psychiatry,* 7 (1), Article 14. <https://doi.org/10.1186/1744-859X-7-14>

Kimura, M., Daibo, I., & Yogo, M. (2008). The study of emotional contagion from the perspective of interpersonal relationships. *Social Behavior and Personality: An International Journal,* 36(1), 27-42. <https://doi.org/10.2224/sbp.2008.36.1.27>

Koku, P. S., & Savas, S. (2016). Restaurant tipping and customers’ susceptibility to emotional contagion. *Journal of Services Marketing,* 30(7), 762-772. <https://doi.org/10.1108/JSM-03-2016-0103>

Kordsachia, C. C., Labuschagne, I., & Stout, J. C. (2018). Visual scanning of the eye region of human faces predicts emotion recognition performance in Huntington’s disease. *Neuropsychology,* 32(3), 356-365. <https://doi.org/10.1037/neu0000424>

Korpal, P., & Jasielska, A. (2019). Investigating interpreters' empathy: Are emotions in simultaneous interpreting contagious? *Target,* 31(1), 2-24. <https://doi.org/10.1075/target.17123.kor>

Kramer, A. D. I., Guillory, J. E., & Hancock, J. T. (2014). Experimental evidence of massive-scale emotional contagion through social networks. P*NAS Proceedings of the National Academy of Sciences of the United States of America,* 111(24), 8788-8790. <https://doi.org/10.1073/pnas.1320040111>

Kuang, B., Li, X., Li, X., Lin, M., Liu, S., & Hu, P. (2021). The effect of eye gaze direction on emotional mimicry: A multimodal study with electromyography and electroencephalography. *NeuroImage,* 226, Article 117604. <https://doi.org/10.1016/j.neuroimage.2020.117604>

Kuang, B., Peng, S., Xie, X., & Hu, P. (2019). Universality vs. cultural specificity in the relations among emotional contagion, emotion regulation, and mood state: An emotion process perspective. *Frontiers in Psychology,* 10, Article 186. <https://doi.org/10.3389/fpsyg.2019.00186>

Laguna, M., Mielniczuk, E., & Gorgievski, M. J. (2021). Business owner-employees contagion of work-related affect and employees’ innovative behavior in small firms. *Applied Psychology: An International Review,* 70(4), 1543-1571. <https://doi.org/10.1111/apps.12288>

Lara, A., Crego, A., & Romero-Maroto, M. (2012). Emotional contagion of dental fear to children: The fathers' mediating role in parental transfer of fear. International *Journal of Paediatric Dentistry,* 22(5), 324-330. <https://doi.org/10.1111/j.1365-263X.2011.01200.x>

Larwood, J. L., & Dingle, G. A. (2022). The effects of emotionally congruent sad music listening in young adults high in rumination. *Psychology of Music,* 50(1), 218-229. <https://doi.org/10.1177/0305735620988793>

Le Blanc, P. M., Bakker, A. B., Peeters, M. C. W., van Heesch, N. C. A., & Schaufeli, W. B. (2001). Emotional job demands and burnout among oncology care providers. *Anxiety, Stress & Coping: An International Journal,* 14(3), 243-263. <https://doi.org/10.1080/10615800108248356>

Lee, M. T., & Theokary, C. (2021). The superstar social media influencer: Exploiting linguistic style and emotional contagion over content? *Journal of Business Research,* 132, 860-871. <https://doi.org/10.1016/j.jbusres.2020.11.014>

Lehmann-Willenbrock, N., Meyers, R. A., Kauffeld, S., Neininger, A., & Henschel, A. (2011). Verbal interaction sequences and group mood: Exploring the role of team planning communication. *Small Group Research,* 42(6), 639-668. <https://doi.org/10.1177/1046496411398397>

Lehmann, A., Bahçesular, K., Brockmann, E.-M., Biederbick, S.-E., Dziobek, I., Gallinat, J., & Montag, C. (2014). Subjective experience of emotions and emotional empathy in paranoid schizophrenia. *Psychiatry Research,* 220(3), 825-833. <https://doi.org/10.1016/j.psychres.2014.09.009>

Li, J., Chen, X.-P., Kotha, S., & Fisher, G. (2017). Catching fire and spreading it: A glimpse into displayed entrepreneurial passion in crowdfunding campaigns. *Journal of Applied Psychology,* 102(7), 1075-1090. <https://doi.org/10.1037/apl0000217>

Li, J., Shi, D., Tumnark, P., & Xu, H. (2020). A system for real-time intervention in negative emotional contagion in a smart classroom deployed under edge computing service infrastructure. *Peer-to-Peer Networking and Applications,* 13(5), 1706-1719. <https://doi.org/10.1007/s12083-019-00863-8>

Li, J., Zhang, J., & Yang, Z. (2017). Associations between a leader's work passion and an employee's work passion: A moderated mediation model. *Frontiers in Psychology,* 8, Article 1447. <https://doi.org/10.3389/fpsyg.2017.01447>

Liang, S.-G., & Chi, S.-C. S. (2013). Transformational leadership and follower task performance: The role of susceptibility to positive emotions and follower positive emotions. *Journal of Business and Psychology,* 28(1), 17-29. <https://doi.org/10.1007/s10869-012-9261-x>

Lima, C. F., Arriaga, P., Anikin, A., Pires, A. R., Frade, S., Neves, L., & Scott, S. K. (2021). Authentic and posed emotional vocalizations trigger distinct facial responses. *Cortex: A Journal Devoted to the Study of the Nervous System and Behavior,* 141, 280-292. <https://doi.org/10.1016/j.cortex.2021.04.015>

Lin, J. S. C., & Lin, C. Y. (2011). What makes service employees and customers smile: Antecedents and consequences of the employees' affective delivery in the service encounter. *Journal of Service Management,* 22(2), 183-201. <https://doi.org/10.1108/09564231111124217>

Lin, X. (2015). How does procedural justice climate influence individual outcomes? An affective perspective. *Asia Pacific Journal of Management,* 32(3), 771-800. <http://dx.doi.org/10.1007/s10490-015-9421-4>

Lin, Y. R., & Margolin, D. (2014). The ripple of fear, sympathy, and solidarity during the Boston bombings. *EPJ Data Science,* 3(1), 1-28. <http://dx.doi.org/10.1140/epjds/s13688-014-0031-z>

Lischetzke, T., Cugialy, M., Apt, T., Eid, M., & Niedeggen, M. (2020). Are Those Who Tend to Mimic Facial Expressions Especially Vulnerable to Emotional Contagion? *Journal of Nonverbal Behavior,* 44(1), 133-152. <http://dx.doi.org/10.1007/s10919-019-00316-z>

Lischke, A., Pahnke, R., Mau-Moeller, A., Jacksteit, R., & Weippert, M. (2020). Sex-specific relationships between interoceptive accuracy and emotion regulation. *Frontiers in Behavioral Neuroscience,* 14, Article 67. <https://doi.org/10.3389/fnbeh.2020.00067>

Lischke, A., Weippert, M., Mau-Moeller, A., Jacksteit, R., & Pahnke, R. (2020). Interoceptive accuracy is associated with emotional contagion in a valence- and sex-dependent manner. *Social Neuroscience,* 15(2), 227-233. <https://doi.org/10.1080/17470919.2019.1690573>

Lishner, D. A., Cooter, A. B., & Zald, D. H. (2008). Rapid emotional contagion and expressive congruence under strong test conditions. *Journal of Nonverbal Behavior*, 32(4), 225-239. <https://doi.org/10.1007/s10919-008-0053-y>

Lishner, D. A., Hong, P. Y., Jiang, L., Vitacco, M. J., & Neumann, C. S. (2015). Psychopathy, narcissism, and borderline personality: A critical test of the affective empathy-impairment hypothesis. *Personality and Individual Differences,* 86, 257-265. <https://doi.org/10.1016/j.paid.2015.05.036>

Lishner, D. A., Vitacco, M. J., Hong, P. Y., Mosley, J., Miska, K., & Stocks, E. L. (2012). Evaluating the relation between psychopathy and affective empathy: Two preliminary studies. *International Journal of Offender Therapy and Comparative Criminology,* 56(8), 1161-1181. <https://doi.org/10.1177/0306624X11421891>

Liu, B., Zhu, N., Wang, H., Li, F., & Men, C. (2021). Protecting nurses from mistreatment by patients: A cross-sectional study on the roles of emotional contagion susceptibility and emotional regulation ability. *International Journal of Environmental Research and Public Health,* 18(12), Article 6331. <https://doi.org/10.3390/ijerph18126331>

Liu, X. Y., De Pater, I. E., & Ilies, R. (2021). Turning lemons into lemonade: The role of proactive personality and information exchange in limiting reciprocal negative affect transference in service encounters. *Journal of Organizational Behavior,* 42(9), 1282-1300. <http://dx.doi.org/10.1002/job.2542>

Lohmann, K., Pyka, S. S., & Zanger, C. (2017). The effects of smileys on receivers’ emotions. *Journal of Consumer Marketing,* 34(6), 489-495. <https://doi.org/10.1108/JCM-02-2017-2120>

Lopes, R. R., Navarro, J., & Silva, A. J. (2018). Emotions as proximal causes of word of mouth: A nonlinear approach. *Nonlinear Dynamics, Psychology, and Life Sciences,* 22(1), 103-125.

Lopes, R. R., Navarro, J., Caetano, A., & Silva, A. J. (2017). Forecasting the influence of customer-related micro-events on employees’ emotional, attitudinal and physiological responses. *European Journal of Work and Organizational Psychology,* 26(6), 779-797. <https://doi.org/10.1080/1359432X.2017.1360286>

Luckhurst, C., Hatfield, E., & Gelvin-Smith, C. (2017). Capacity for empathy and emotional contagion in those with psychopathic personalities. *Interpersona*, 11(1), 70-91. <https://doi.org/10.5964/ijpr.v11i1.247>

Lundqvist, L.-O. (1995). Facial EMG reactions to facial expressions: A case of facial emotional contagion? *Scandinavian Journal of Psychology*, 36(2), 130-141. <https://doi.org/10.1111/j.1467-9450.1995.tb00974.x>

Lundqvist, L.-O. (2008). The relationship between the Biosocial Model of Personality and susceptibility to emotional contagion: A structural equation modeling approach. *Personality and Individual Differences,* 45(1), 89-95. <https://doi.org/10.1016/j.paid.2008.03.003>

Lundqvist, L.-O., & Dimberg, U. (1995). Facial expressions are contagious. *Journal of Psychophysiology,* 9(3), 203-211.

Magen, E., & Konasewich, P. A. (2011). Women support providers are more susceptible than men to emotional contagion following brief supportive interactions. P*sychology of Women Quarterly,* 35(4), 611-616. <https://doi.org/10.1177/0361684311423912>

Manera, V., Grandi, E., & Colle, L. (2013). Susceptibility to emotional contagion for negative emotions improves detection of smile authenticity. *Frontiers in Human Neuroscience,* 7, Article 6. <http://dx.doi.org/10.3389/fnhum.2013.00006>

Manini, B., Cardone, D., Ebisch, S. J. H., Bafunno, D., Aureli, T., & Merla, A. (2013). Mom feels what her child feels: Thermal signatures of vicarious autonomic response while watching children in a stressful situation. *Frontiers in Human Neuroscience,* 7, Article 299. <https://doi.org/10.3389/fnhum.2013.00299>

Marshall, C. R., Hardy, C. J. D., Russell, L. L., Bond, R. L., Sivasathiaseelan, H., Greaves, C., Moore, K. M., Agustus, J. L., van Leeuwen, J. E. P., Wastling, S. J., Rohrer, J. D., Kilner, J. M., & Warren, J. D. (2019). The functional neuroanatomy of emotion processing in frontotemporal dementias. *Brain: A Journal of Neurology,* 142(9), 2873-2887. <https://doi.org/10.1093/brain/awz204>

Mauersberger, H., & Hess, U. (2019). When smiling back helps and scowling back hurts: Individual differences in emotional mimicry are associated with self-reported interaction quality during conflict interactions. *Motivation and Emotion,* 43(3), 471-482. <https://doi.org/10.1007/s11031-018-9743-x>

Mauersberger, H., Blaison, C., Kafetsios, K., Kessler, C. L., & Hess, U. (2015). Individual differences in emotional mimicry: Underlying traits and social consequences. *European Journal of Personality,* 29(5), 512-529. <https://doi.org/10.1002/per.2008>

Mazzuca, S., Kafetsios, K., Livi, S., & Presaghi, F. (2019). Emotion regulation and satisfaction in long-term marital relationships: The role of emotional contagion. *Journal of Social and Personal Relationships,* 36(9), 2880-2895. <https://doi.org/10.1177/0265407518804452>

McGettigan, C., Walsh, E., Jessop, R., Agnew, Z. K., Sauter, D. A., Warren, J. E., & Scott, S. K. (2015). Individual differences in laughter perception reveal roles for mentalizing and sensorimotor systems in the evaluation of emotional authenticity. *Cerebral Cortex,* 25(1), 246-257. <https://doi.org/10.1093/cercor/bht227>

McIntosh, D. N. (2006). Spontaneous facial mimicry, liking and emotional contagion. *Polish Psychological Bulletin,* 37(1), 31-42.

Meier, I. M., Bos, P. A., Hamilton, K., Stein, D. J., van Honk, J., & Malcolm-Smith, S. (2016). Naltrexone increases negatively-valenced facial responses to happy faces in female participants. *Psychoneuroendocrinology,* 74, 65-68. <https://doi.org/10.1016/j.psyneuen.2016.08.022>

Miller, K., Birkholt, M., Scott, C., & Stage, C. (1995). Empathy and burnout in human service work: An extension of a communication model. *Communication Research,* 22(2), 123-147. <https://doi.org/10.1177/009365095022002001>

Minio-Paluello, I., Porciello, G., Gandolfo, M., Boukarras, S., & Aglioti, S. M. (2020). The enfacement illusion boosts facial mimicry. *Cortex: A Journal Devoted to the Study of the Nervous System and Behavior,* 123, 113-123. <https://doi.org/10.1016/j.cortex.2019.10.001>

Mireault, G. C., Crockenberg, S. C., Sparrow, J. E., Cousineau, K., Pettinato, C., & Woodard, K. (2015). Laughing matters: Infant humor in the context of parental affect. *Journal of Experimental Child Psychology,* 136, 30-41. <https://doi.org/10.1016/j.jecp.2015.03.012>

Moody, E. J., McIntosh, D. N., Mann, L. J., & Weisser, K. R. (2007). More than mere mimicry? The influence of emotion on rapid facial reactions to faces. *Emotion,* 7(2), 447-457. <https://doi.org/10.1037/1528-3542.7.2.447>

Moore, M., Shafer, A. T., Bakhtiari, R., Dolcos, F., & Singhal, A. (2019). Integration of spatio-temporal dynamics in emotion-cognition interactions: A simultaneous fMRI-ERP investigation using the emotional oddball task. *NeuroImage,* 202, Article 116078. <https://doi.org/10.1016/j.neuroimage.2019.116078>

Moskowitz, S., & Dewaele, J. M. (2021). Is teacher happiness contagious? A study of the link between perceptions of language teacher happiness and student attitudes. *Innovation in Language Learning and Teaching,* 15(2), 117-130. <https://doi.org/10.1080/17501229.2019.1707205>

Moura, R., Camilo, C., & Luís, S. (2021). As strong as we are united: Effects of intrapersonal and interpersonal emotion regulation on quality of life in women with breast cancer. *Frontiers in Psychology,* 12, Article 661496. <https://doi.org/10.3389/fpsyg.2021.661496>

Mui, P. H. C., Goudbeek, M. B., Roex, C., Spierts, W., & Swerts, M. G. J. (2018). Smile mimicry and emotional contagion in audio-visual computer-mediated communication. *Frontiers in Psychology,* 9, Article 2077. <https://doi.org/10.3389/fpsyg.2018.02077>

Muszynski, M., Kostoulas, T., Lombardo, P., Pun, T., & Chanel, G. (2018). Aesthetic highlight detection in movies based on synchronization of spectators' reactions. *ACM Transactions on Multimedia Computing, Communications, and Applications,* 14(3), 1-23. <https://doi.org/10.1145/3175497>

Mutic, S., Parma, V., Brünner, Y. F., & Freiherr, J. (2016). You smell dangerous: Communicating fight responses through human chemosignals of aggression. *Chemical Senses,* 41(1), 35-43. <https://doi.org/10.1093/chemse/bjv058>

Neumann, R., & Strack, F. (2000). Mood contagion: The automatic transfer of mood between persons. *Journal of Personality and Social Psychology,* 79(2), 211-223. <https://doi.org/10.1037/0022-3514.79.2.211>

Neves, L., Cordeiro, C., Scott, S. K., Castro, S. L., & Lima, C. F. (2018). High emotional contagion and empathy are associated with enhanced detection of emotional authenticity in laughter. *The Quarterly Journal of Experimental Psychology,* 71(11), 2355-2363. <https://doi.org/10.1177/1747021817741800>

Niedtfeld, I. (2017). Experimental investigation of cognitive and affective empathy in borderline personality disorder: Effects of ambiguity in multimodal social information processing. *Psychiatry Research,* 253, 58-63. <https://doi.org/10.1016/j.psychres.2017.03.037>

Nilsonne, G., Tamm, S., Golkar, A., Sörman, K., Howner, K., Kristiansson, M., Olsson, A., Ingvar, M., & Petrovic, P. (2017). Effects of 25 mg oxazepam on emotional mimicry and empathy for pain: A randomized controlled experiment. *Royal Society Open Science,* 4(3), Article 160607. <https://doi.org/10.1098/rsos.160607>

Northington, W. M., Gillison, S. T., Beatty, S. E., & Vivek, S. (2021). I don't want to be a rule enforcer during the COVID-19 pandemic: Frontline employees' plight. *Journal of Retailing and Consumer Services,* 63, Article 102723. <https://doi.org/10.1016/j.jretconser.2021.102723>

Nummenmaa, L., Hirvonen, J., Parkkola, R., & Hietanen, J. K. (2008). Is emotional contagion special? An fMRI study on neural systems for affective and cognitive empathy. *NeuroImage,* 43(3), 571-580. <https://doi.org/10.1016/j.neuroimage.2008.08.014>

Nyquist, E., Allen, J., & Erks, R. (2018). When the boss came to the meeting...: Hierarchical distance and emotional labor in workplace meetings. *Consulting Psychology Journal: Practice and Research,* 70(3), 207-226. <https://doi.org/10.1037/cpb0000111>

O’Neill, D. F. (2008). Injury contagion in alpine ski racing: The effect of injury on teammates’ performance. *Journal of Clinical Sport Psychology,* 2(3), 278-292. <https://doi.org/10.1123/jcsp.2.3.278>

Oberman, L. M., Winkielman, P., & Ramachandran, V. S. (2009). Slow echo: Facial EMG evidence for the delay of spontaneous, but not voluntary, emotional mimicry in children with autism spectrum disorders. *Developmental Science,* 12(4), 510-520. <https://doi.org/10.1111/j.1467-7687.2008.00796.x>

Olszanowski, M., Lewandowska, P., Ozimek, A., & Frankowska, N. (2022). The effect of facial self-resemblance on emotional mimicry. *Journal of Nonverbal Behavior.* 46(2), 197-213. <https://doi.org/10.1007/s10919-021-00395-x>

Olszanowski, M., Wróbel, M., & Hess, U. (2020). Mimicking and sharing emotions: A re-examination of the link between facial mimicry and emotional contagion. *Cognition and Emotion,* 34(2), 367-376. <https://doi.org/10.1080/02699931.2019.1611543>

Omdahl, B. L., & O'Donnell, C. (1999). Emotional contagion, empathic concern, and communicative responsiveness as variables affecting nurses' stress and occupational commitment. *Journal of Advanced Nursing,* 29(6), 1351-1359. <https://doi.org/10.1046/j.1365-2648.1999.01021.x>

Owens, E. S., McPharlin, F. W. H., Brooks, N., & Fritzon, K. (2018). The effects of empathy, emotional intelligence and psychopathy on interpersonal interactions. *Psychiatry, Psychology and Law,* 25(1), 2022-01-18 00:00:00. <https://doi.org/10.1080/13218719.2017.1347936>

Panger, G. (2018). People tend to wind down, not up, when they browse social media. *Proceedings of the ACM on Human-Computer Interaction,* 2(CSCW), 1-29. <https://doi.org/10.1145/3274402>

Papousek, I., Freudenthaler, H. H., & Schulter, G. (2008). The interplay of perceiving and regulating emotions in becoming infected with positive and negative moods. *Personality and Individual Differences,* 45(6), 463-467. <https://doi.org/10.1016/j.paid.2008.05.021>

Papousek, I., Freudenthaler, H. H., & Schulter, G. (2011). Typical performance measures of emotion regulation and emotion perception and frontal EEG asymmetry in an emotional contagion paradigm. *Personality and Individual Differences,* 51(8), 1018-1022. <https://doi.org/10.1016/j.paid.2011.08.013>

Papousek, I., Reiser, E. M., Weber, B., Freudenthaler, H. H., & Schulter, G. (2012). Frontal brain asymmetry and affective flexibility in an emotional contagion paradigm. *Psychophysiology,* 49(4), 489-498. <https://doi.org/10.1111/j.1469-8986.2011.01324.x>

Papousek, I., Ruch, W., Freudenthaler, H. H., Kogler, E., Lang, B., & Schulter, G. (2009). Gelotophobia, emotion-related skills and responses to the affective states of others. *Personality and Individual Differences,* 47(1), 58-63. <https://doi.org/10.1016/j.paid.2009.01.047>

Park, S., Choi, S. J., Mun, S., & Whang, M. (2019). Measurement of emotional contagion using synchronization of heart rhythm pattern between two persons: Application to sales managers and sales force synchronization. *Physiology & Behavior,* 200, 148-158.

Peled-Avron, L., Levy-Gigi, E., Richter-Levin, G., Korem, N., & Shamay-Tsoory, S. G. (2016). The role of empathy in the neural responses to observed human social touch. *Cognitive, Affective, & Behavioral Neuroscience,* 16(5), 802-813. <https://doi.org/10.3758/s13415-016-0432-5>

Peng, S., Kuang, B., & Hu, P. (2020). Right temporoparietal junction modulates in-group bias in facial emotional mimicry: A tDCS study. *Frontiers in Behavioral Neuroscience,* 14(10), Article 143. <https://doi.org/10.3389/fnbeh.2020.00143>

Peng, S., Kuang, B., Zhang, L., & Hu, P. (2021). Right temporoparietal junction plays a role in the modulation of emotional mimicry by group membership. *Frontiers in Human Neuroscience,* 15, Article 606292. <https://doi.org/10.3389/fnhum.2021.606292>

Peng, S., Zhang, L., & Hu, P. (2021). Relating self–other overlap to ingroup bias in emotional mimicry. *Social Neuroscience,* 16(4), 439-447. <https://doi.org/10.1080/17470919.2021.1940273>

Petitta, L., & Jiang, L. (2019). Burning out? Watch your own incivility and the emotions you spread. *Work: Journal of Prevention, Assessment & Rehabilitation,* 64(4), 671-683. <https://doi.org/10.3233/WOR-193029>

Petitta, L., & Jiang, L. (2020a). How emotional contagion relates to burnout: A moderated mediation model of job insecurity and group member prototypicality. *International Journal of Stress Management,* 27(1), 12-22. <https://doi.org/10.1037/str0000134>

Petitta, L., & Jiang, L. (2020b). How group goal setting mediates the link between individual‐level emotion‐related factors and team performance. *Journal of Theoretical Social Psychology,* 4(1), 3-20. <https://doi.org/10.1002/jts5.54>

Petitta, L., Jiang, L., & Härtel, C. E. J. (2017). Emotional contagion and burnout among nurses and doctors: Do joy and anger from different sources of stakeholders matter? *Stress and Health: Journal of the International Society for the Investigation of Stress,* 33(4), 358-369. <https://doi.org/10.1002/smi.2724>

Petitta, L., Probst, T. M., Ghezzi, V., & Barbaranelli, C. (2019). Cognitive failures in response to emotional contagion: Their effects on workplace accidents. *Accident Analysis and Prevention,* 125, 165-173. <https://doi.org/10.1016/j.aap.2019.01.018>

Petitta, L., Probst, T. M., Ghezzi, V., & Barbaranelli, C. (2020). Economic stress, emotional contagion and safety outcomes: A cross-country study. *Work: Journal of Prevention, Assessment & Rehabilitation,* 66(2), 421-435. <https://doi.org/10.3233/WOR-203182>

Petitta, L., Probst, T. M., Ghezzi, V., & Barbaranelli, C. (2021a). Emotional contagion as a trigger for moral disengagement: Their effects on workplace injuries. *Safety Science,* 140, Article 105317. <https://doi.org/10.1016/j.ssci.2021.105317>

Petitta, L., Probst, T. M., Ghezzi, V., & Barbaranelli, C. (2021b). The impact of emotional contagion on workplace safety: Investigating the roles of sleep, health, and production pressure. *Current Psychology: A Journal for Diverse Perspectives on Diverse Psychological Issues.*(42)3, 2362-2376. <https://doi.org/10.1007/s12144-021-01616-8>

Pinilla, A., Tamayo, R. M., & Neira, J. (2020). How do induced affective states bias emotional contagion to faces? A three-dimensional model. *Frontiers in Psychology,* 11, Article 97. <https://doi.org/10.3389/fpsyg.2020.00097>

Pizarro-Campagna, E., Terrett, G., Jovev, M., Rendell, P. G., Henry, J. D., & Chanen, A. M. (2020). Rapid facial mimicry responses are preserved in youth with first presentation borderline personality disorder. *Journal of Affective Disorders,* 266, 14-21. <https://doi.org/10.1016/j.jad.2020.01.097>

Preston, S. D., & Stansfield, R. B. (2008). I know how you feel: Task-irrelevant facial expressions are spontaneously processed at a semantic level. *Cognitive, Affective & Behavioral Neuroscience,* 8(1), 54-64. <https://doi.org/10.3758/CABN.8.1.54>

Pugh, S. D. (2001). Service with a smile: Emotional contagion in the service encounter. *Academy of Management Journal,* 44(5), 1018-1027. <https://doi.org/10.2307/3069445>

Quintana, P., Nolet, K., Baus, O., & Bouchard, S. (2019). The effect of exposure to fear-related body odorants on anxiety and interpersonal trust toward a virtual character. *Chemical Senses,* 44(9), 683-692. <https://doi.org/10.1093/chemse/bjz063>

Ramanathan, S., & McGill, A. L. (2007). Consuming with others: Social influences on moment-to-moment and retrospective evaluations of an experience. *Journal of Consumer Research,* 34(4), 506-524. <https://doi.org/10.1086/520074>

Reddy, R. P., Mathulla, A. R., & Rajeswaran, J. (2022). A pilot study of perspective taking and emotional contagion in mental health professionals: Glass brain view of empathy. *Indian Journal of Psychological Medicine,* 44(1), 53-58. <https://doi.org/10.1177/0253717620973380>

Rempala, D. M. (2012). Interhemispheric communication and emotional reactivity. *Current Research in Social Psychology,* 19(2), 10-24.

Rempala, D. M. (2013). Cognitive strategies for controlling emotional contagion. *Journal of Applied Social Psychology,* 43(7), 1528-1537. <https://doi.org/10.1111/jasp.12146>

Rhee, S. Y., Park, H., & Bae, J. (2020). Network structure of affective communication and shared emotion in teams. *Behavioral Sciences,* 10(10), Article 159. <https://doi.org/10.3390/bs10100159>

Rosenbusch, H., Evans, A. M., & Zeelenberg, M. (2019). Multilevel emotion transfer on YouTube: Disentangling the effects of emotional contagion and homophily on video audiences. *Social Psychological and Personality Science*, 10(8), 1028-1035. <https://doi.org/10.1177/1948550618820309>

Rosner, R., Beutler, L. E., & Daldrup, R. J. (2000). Vicarious emotional experience and emotional expression in group psychotherapy. *Journal of Clinical Psychology,* 56(1). [https://doi.org/10.1002/(SICI)1097-4679(200001)56:1<1::AID-JCLP1>3.0.CO;2-7](https://doi.org/10.1002/(SICI)1097-4679(200001)56:1%3c1::AID-JCLP1%3e3.0.CO;2-7)

Rueff-Lopes, R., Navarro, J., Caetano, A., & Silva, A. J. (2015). A Markov chain analysis of emotional exchange in voice-to-voice communication: Testing for the mimicry hypothesis of emotional contagion. *Human Communication Research,* 41(3), 412-434. <https://doi.org/10.1111/hcre.12051>

Ruffman, T., Then, R., Cheng, C., & Imuta, K. (2019). Lifespan differences in emotional contagion while watching emotion-eliciting videos. *PLoS ONE,* 14(1), Article e0209253. <http://dx.doi.org/10.1371/journal.pone.0209253>

Ruiz, P., Pilatti, A., & Pautassi, R. M. (2020). Consequences of alcohol use, and its association with psychological distress, sensitivity to emotional contagion and age of onset of alcohol use, in Uruguayan youth with or without college degree. *Alcohol,* 82, 91-101. <https://doi.org/10.1016/j.alcohol.2019.09.001>

Rumbold, J. L., Newman, J. A., Foster, D., Rhind, D. J. A., Phoenix, J., & Hickey, L. (2021). Assessing post-game emotions in soccer teams: The role of distinct emotional dynamics. *European Journal of Sport Science.* 22(6), 888-896. <https://doi.org/10.1080/17461391.2021.1916079>

Rushby, J. A., McDonald, S., Randall, R., de Sousa, A., Trimmer, E., & Fisher, A. (2013). Impaired emotional contagion following severe traumatic brain injury. *International Journal of Psychophysiology,* 89(3), 466-474. <https://doi.org/10.1016/j.ijpsycho.2013.06.013>

Sachisthal, M. S. M., Sauter, D. A., & Fischer, A. H. (2016). Mimicry of ingroup and outgroup emotional expressions. *Comprehensive Results in Social Psychology,* 1(1-2), 86-105. <https://doi.org/10.1080/23743603.2017.1298355>

Salvadori, E. A., Colonnesi, C., Vonk, H. S., Oort, F. J., & Aktar, E. (2021). Infant emotional mimicry of strangers: Associations with parent emotional mimicry, parent-infant mutual attention, and parent dispositional affective empathy. *International Journal of Environmental Research and Public Health,* 18(2), Article 654. <https://doi.org/10.3390/ijerph18020654>

Scambler, D. J., Hepburn, S., Rutherford, M. D., Wehner, E. A., & Rogers, S. J. (2007). Emotional responsivity in children with autism, children with other developmental disabilities, and children with typical development. *Journal of Autism and Developmental Disorders,* 37(3), 553-563. <https://doi.org/10.1007/s10803-006-0186-y>

Schei, G. S., Haugen, T., Jones, G., Sæther, S. A., & Høigaard, R. (2021). A qualitative exploration of collective collapse in a Norwegian qualifying Premier League soccer match: The successful team's perspective. *Frontiers in Psychology,* 12, Article 777597. <https://doi.org/10.3389/fpsyg.2021.777597>

Schoner-Schatz, L., Hofmann, V., & Stokburger-Sauer, N. E. (2021). Destination's social media communication and emotions: An investigation of visit intentions, word-of-mouth and travelers’ facially expressed emotions. *Journal of Destination Marketing and Management,* 22, Article 100661. <https://doi.org/10.1016/j.jdmm.2021.100661>

Shaw, P. V., Wilson, G. A., & Antony, M. M. (2020). Examination of emotional contagion and social anxiety using novel video stimuli. *Anxiety, Stress & Coping: An International Journal.* 34(2), 215-227. <https://doi.org/10.1080/10615806.2020.1839729>

Silas, H. J., Binfet, J.-T., & Ford, A. T. (2019). Therapeutic for all? Observational assessments of therapy canine stress in an on-campus stress-reduction program. *Journal of Veterinary Behavior: Clinical Applications and Research*, 32, 6-13. <https://doi.org/10.1016/j.jveb.2019.03.009>

Skinner, A. L., Osnaya, A., Patel, B., & Perry, S. P. (2020). Mimicking others’ nonverbal signals is associated with increased attitude contagion. *Journal of Nonverbal Behavior,* 44(1), 117-131. <https://doi.org/10.1007/s10919-019-00322-1>

Slapničar, S., Ličen, M., Hartmann, F. G. H., Ozimič, A. S., & Repovš, G. (2021). Management accountants’ empathy and their violation of fiduciary duties: A replication and extension study using fMRI. *Behavioral Research in Accounting,* 33(1), 21-42. <https://doi.org/10.2308/BRIA-2020-021>

Smirnov, D., Saarimäki, H., Glerean, E., Hari, R., Sams, M., & Nummenmaa, L. (2019). Emotions amplify speaker–listener neural alignment. *Human Brain Mapping*, 40(16), 4777-4788. <https://doi.org/10.1002/hbm.24736>

Söderlund, M., & Rosengren, S. (2007). Receiving word-of-mouth from the service customer: An emotion-based effectiveness assessment. *Journal of Retailing and Consumer Services,* 14(2), 123-136. <https://doi.org/10.1016/j.jretconser.2006.10.001>

Sonnby-Borgström, M. (2009). Alexithymia as related to facial imitation, mentalization, empathy, and internal working models-of-self and -others. *Neuropsychoanalysis,* 11(1), 111-128. <https://doi.org/10.1080/15294145.2009.10773602>

Sonnby-Borgström, M., & Jõnsson, P. (2004). Dismissing-avoidant pattern of attachment and mimicry reactions at different levels of information processing. *Scandinavian Journal of Psychology,* 45(2), 103-113. <https://doi.org/10.1111/j.1467-9450.2004.00385.x>

Sonnby-Borgström, M., Jönsson, P., & Svensson, O. (2008a). Imitative responses and verbally reported emotional contagion from spontaneous, unconscious to emotionally regulated, conscious information-processing levels. *Neuropsychoanalysis,* 10(1), 81-98. <https://doi.org/10.1080/15294145.2008.10773573>

Sonnby-Borgström, M., Jönsson, P., & Svensson, O. (2008b). Gender differences in facial imitation and verbally reported emotional contagion from spontaneous to emotionally regulated processing levels. *Scandinavian Journal of Psychology,* 49(2), 111-122. <https://doi.org/10.1111/j.1467-9450.2008.00626.x>

Soussignan, R., Dollion, N., Schaal, B., Durand, K., Reissland, N., & Baudouin, J.-Y. (2018). Mimicking emotions: How 3-12-month-old infants use the facial expressions and eyes of a model. *Cognition & Emotion,* 32(4), 827-842. <https://doi.org/10.1080/02699931.2017.1359015>

Stavrova, O., & Meckel, A. (2017). Perceiving emotion in non-social targets: The effect of trait empathy on emotional contagion through art. *Motivation and Emotion,* 41(4), 492-509. <https://doi.org/10.1007/s11031-017-9619-5>

Sturm, V. E., Yokoyama, J. S., Seeley, W. W., Kramer, J. H., Miller, B. L., & Rankin, K. P. (2013). Heightened emotional contagion in mild cognitive impairment and Alzheimer’s disease is associated with temporal lobe degeneration. *PNAS Proceedings of the National Academy of Sciences of the United States of America,* 110(24), 9944-9949. <http://dx.doi.org/10.1073/pnas.1301119110>

Surakka, V., & Hietanen, J. K. (1998). Facial and emotional reactions to Duchenne and non-Duchenne smiles. *International Journal of Psychophysiology,* 29(1), 23-33. <http://dx.doi.org/10.1016/S0167-8760(97)00088-3>

Sy, T., & Choi, J. N. (2013). Contagious leaders and followers: Exploring multi-stage mood contagion in a leader activation and member propagation (LAMP) model. *Organizational Behavior and Human Decision Processes,* 122(2), 127-140. <http://dx.doi.org/10.1016/j.obhdp.2013.06.003>

Tamborini, R., Salomonson, K., & Bahk, C. (1993). The relationship of empathy to comforting behavior following film exposure. *Communication Research,* 20(5), 723-738. <https://doi.org/10.1177/009365093020005005>

Tamietto, M., Castelli, L., Vighetti, S., Perozzo, P., Geminiani, G., Weiskrantz, L., & de Gelder, B. (2009). Unseen facial and bodily expressions trigger fast emotional reactions. *PNAS Proceedings of the National Academy of Sciences of the United States of America,* 106(42), 17662-17666. <https://doi.org/10.1073/pnas.0908994106>

Tamm, S., Schwarz, J., Thuné, H., Kecklund, G., Petrovic, P., Åkerstedt, T., Fischer, H., Lekander, M., & Nilsonne, G. (2020). A combined fMRI and EMG study of emotional contagion following partial sleep deprivation in young and older humans. *Scientific Reports,* 10(1), Article 17944. <https://doi.org/10.1038/s41598-020-74489-9>

Tang, J., Yu, G., & Yao, X. (2021). Emotional contagion in the online depression community. *Healthcare (Switzerland),* 9(12), Article 1609. <https://doi.org/10.3390/healthcare9121609>

Thibault, C., Gabriel, D., Comte, A., Haffen, E., Moulin, T., & Pazart, L. (2022). Interbrain emotional connection during music performances is driven by physical proximity and individual traits. *Annals of the New York Academy of Sciences*, 1508(1), 178-195. <https://doi.org/10.1111/nyas.14711>

Totterdell, P. (2000). Catching moods and hitting runs: mood linkage and subjective performance in professional sport teams. *Journal of Applied Psychology,* 85(6), 848-859. <https://doi.org/10.1037/0021-9010.85.6.848>

Trautmann, S., Kräplin, A., Dieterich, R., Richter, J., & Muehlhan, M. (2018). The role of childhood trauma and stress reactivity for increased alcohol craving after induced psychological trauma: An experimental analogue study. *Psychopharmacology*, 235(10), 2883-2895. <https://doi.org/10.1007/s00213-018-4979-4>

Trautmann, S., Reineboth, M., Trikojat, K., Richter, J., Hagenaars, M. A., Kanske, P., & Schäfer, J. (2018). Susceptibility to others' emotions moderates immediate self-reported and biological stress responses to witnessing trauma. *Behaviour Research and Therapy,* 110, 55-63. <https://doi.org/10.1016/j.brat.2018.09.001>

Tsai, J., Bowring, E., Marsella, S., Wood, W., & Tambe, M. (2021). A study of emotional contagion with virtual characters. *Intelligent Virtual Agents,* 81-88.

Umasuthan, H., Park, O. J., & Ryu, J. H. (2017). Influence of empathy on hotel guests’ emotional service experience. *Journal of Services Marketing,* 31(6), 618-635. <https://doi.org/10.1108/JSM-06-2016-0220>

Ustrov, Y., Valverde, M., & Ryan, G. (2016). Insights into emotional contagion and its effects at the hotel front desk. International *Journal of Contemporary Hospitality Management,* 28(10), 2285-2309. <http://dx.doi.org/10.1108/IJCHM-08-2014-0378>

van der Schalk, J., Fischer, A., Doosje, B., Wigboldus, D., Hawk, S., Rotteveel, M., & Hess, U. (2011). Convergent and divergent responses to emotional displays of ingroup and outgroup. *Emotion,* 11(2), 286-298. <https://doi.org/10.1037/a0022582>

Vannini, N., Enz, S., Sapouna, M., Wolke, D., Watson, S., Woods, S., Dautenhahn, K., Hall, L., Paiva, A., André, E., Aylett, R., & Schneider, W. (2011). “Fearnot!”: A computer-based anti-bullying-programme designed to foster peer intervention. *European Journal of Psychology of Education,* 26(1), 21-44. <https://doi.org/10.1007/s10212-010-0035-4>

Varni, G., Hupont, I., Clavel, C., & Chetouani, M. (2020). Computational study of primitive emotional contagion in dyadic interactions. *IEEE Transactions on Affective Computing,* 11(2), 258-271. <https://doi.org/10.1109/TAFFC.2017.2778154>

Verbeke, W. (1997). Individual differences in emotional contagion of salespersons: Its effect on performance and burnout. *Psychology & Marketing,* 14(6), 617-636. [http://dx.doi.org/10.1002/(SICI)1520-6793(199709)14:6<617::AID-MAR6>3.0.CO;2-A](http://dx.doi.org/10.1002/(SICI)1520-6793(199709)14:6%3c617::AID-MAR6%3e3.0.CO;2-A)

Visser, V. A., van Knippenberg, D., van Kleef, G. A., & Wisse, B. (2013). How leader displays of happiness and sadness influence follower performance: Emotional contagion and creative versus analytical performance. *The Leadership Quarterly,* 24(1), 172-188. <http://dx.doi.org/10.1016/j.leaqua.2012.09.003>

Wang, T. R., & Schrodt, P. (2010). Are emotional intelligence and contagion moderators of the association between students' perceptions of instructors' nonverbal immediacy cues and students' affect? *Communication Reports,* 23(1), 26-38. <http://dx.doi.org/10.1080/08934211003598775>

Wang, X., & Lee, E. W. J. (2021). Negative emotions shape the diffusion of cancer tweets: Toward an integrated social network–text analytics approach. *Internet Research,* 31(2), 401-418. <http://dx.doi.org/10.1108/INTR-04-2020-0181>

Weber, M., & Quiring, O. (2019). Is it really that funny? Laughter, emotional contagion, and heuristic processing during shared media use. *Media Psychology,* 22(2), 173-195. <https://doi.org/10.1080/15213269.2017.1302342>

Wergin, V. V., Mallett, C. J., Mesagno, C., Zimanyi, Z., & Beckmann, J. (2019). When you watch your team fall apart - Coaches' and sport psychologists' perceptions on causes of collective sport team collapse. *Frontiers in Psychology,* 10, Article 1331. <http://dx.doi.org/10.3389/fpsyg.2019.01331>

Wergin, V. V., Zimanyi, Z., Mesagno, C., & Beckmann, J. (2018). When suddenly nothing works anymore within a team - Causes of collective sport team collapse. *Frontiers in Psychology,* 9, Article 2115. <http://dx.doi.org/10.3389/fpsyg.2018.02115>

Wild, B., Erb, M., & Bartels, M. (2001). Are emotions contagious? Evoked emotions while viewing emotionally expressive faces: Quality, quantity, time course, and gender differences. *Psychiatry Research,* 102(2), 109-124. <https://doi.org/10.1016/S0165-1781(01)00225-6>

Woo, K.-s., & Chan, B. (2020). “Service with a smile” and emotional contagion: A replication and extension study. *Annals of Tourism Research,* 80, Article 102850. <https://doi.org/10.1016/j.annals.2019.102850>

Wróbel, M., & Olszanowski, M. (2019). Emotional reactions to dynamic morphed facial expressions: A new method to induce emotional contagion. *Roczniki Psychologiczne,* 22(1), 91-102. <https://doi.org/10.18290/rpsych.2019.22.1-6>

Wróbel, M., Piórkowska, M., Rzeczkowska, M., Troszczyńska, A., Tołopiło, A., & Olszanowski, M. (2021). The “big two” and socially induced emotions: Agency and communion jointly influence emotional contagion and emotional mimicry. *Motivation and Emotion.* 45(5), 683-704. <https://doi.org/10.1007/s11031-021-09897-z>

Wu, T.-J., & Wu, Y. J. (2019). Innovative work behaviors, employee engagement, and surface acting: A delineation of supervisor-employee emotional contagion effects. *Management Decision,* 57(11), 3200-3216. <http://dx.doi.org/10.1108/MD-02-2018-0196>

Wu, T.-Y., & Hu, C. (2009). Abusive supervision and employee emotional exhaustion: Dispositional antecedents and boundaries. *Group & Organization Management,* 34(2), 143-169. <http://dx.doi.org/10.1177/1059601108331217>

Xerri, M. J., Brunetto, Y., Farr-Wharton, B., & Cully, A. (2020). Shaping emotional contagion in healthcare: The role of human resource practices and work harassment. *Personnel Review,* 50, 1665-1684. <https://doi.org/10.1108/PR-06-2020-0484>

Xiao, Z.-M., Lee, M.-H., & Wang, H.-C. (2020). Service innovation and mental health: The multilevel moderating role of group emotional contagion. *Social Behavior and Personality: An International Journal,* 47(10), Article e8143. <http://dx.doi.org/10.2224/sbp.8143>

Xiong, X., Li, Y., Qiao, S., Han, N., Wu, Y., Peng, J., & Li, B. (2018). An emotional contagion model for heterogeneous social media with multiple behaviors. *Physica A: Statistical Mechanics and its Applications,* 490, 185-202. <http://dx.doi.org/10.1016/j.physa.2017.08.025>

Yamashita, Y., & Yamamoto, T. (2021). Perceiving positive facial expression can relieve depressive moods: The effect of emotional contagion on mood in people with subthreshold depression. *Frontiers in Psychology,* 12, Article 535980. <http://dx.doi.org/10.3389/fpsyg.2021.535980>

Yong, M. H., & Ruffman, T. (2014). Emotional contagion: Dogs and humans show a similar physiological response to human infant crying. *Behavioural Processes,* 108, 155-165. <https://doi.org/10.1016/j.beproc.2014.10.006>

Zhang, L., Chen, W., Liu, M., Ou, Y., Xu, E., & Hu, P. (2021). Light makeup decreases receivers’ negative emotional experience. *Scientific Reports,* 11(1), Article 23802. <https://doi.org/10.1038/s41598-021-03129-7>

Zheng, W., Yu, A., Fang, P., & Peng, K. (2020). Exploring collective emotion transmission in face-to-face interactions. *PLoS ONE,* 15(8), 11. <http://dx.doi.org/10.1371/journal.pone.0236953>

Zhou, Y., & Fischer, M. H. (2018). Mimicking non-verbal emotional expressions and empathy development in simulated consultations: An experimental feasibility study. *Patient Education and Counseling,* 101(2), 304-309. <http://dx.doi.org/10.1016/j.pec.2017.08.016>
